# Rhythmic Density Affects Listeners' Emotional Response to Microtiming

**DOI:** 10.3389/fpsyg.2017.01709

**Published:** 2017-10-12

**Authors:** Olivier Senn, Claudia Bullerjahn, Lorenz Kilchenmann, Richard von Georgi

**Affiliations:** ^1^School of Music, Lucerne University of Applied Sciences and Arts, Lucerne, Switzerland; ^2^Department of Social Sciences and Cultural Studies, Institute of Musicology and Music Education, Justus-Liebig-University Giessen, Giessen, Germany; ^3^Media Psychology Department, SRH Hochschule der Populären Künste, Berlin, Germany

**Keywords:** groove, microtiming, emotions, rhythmic density, popular music

## Abstract

Microtiming has been assumed to be vital for the experience of groove, but past research presented conflicting results: some studies found that microtiming is irrelevant for groove, others reported that microtiming has a detrimental effect on the groove experience, yet others described circumstances under which microtiming has no negative impact on groove. The three studies in this paper aim at explaining some of these discrepancies by clarifying to what extent listeners' emotional responses to microtiming depend on the distribution of microtiming deviations across instrumental parts (voicing) or other moderating factors like tempo or rhythmic density. The studies use data from two listening experiments involving expert bass and drums duo recordings in swing and funk style.

– *Study A* investigates the effect of fixed time displacements within and between the parts played by different musicians. Listeners (*n* = 160) reacted negatively to irregularities within the drum track, but the mutual displacement of bass vs. drums did not have an effect.
– *Study B* develops three metrics to calculate the average microtiming magnitude in a musical excerpt. The experiment showed that listeners' (*n* = 160) emotional responses to expert performance microtiming aligned with each other across styles, when microtiming magnitude was adjusted for rhythmic density. This indicates that rhythmic density is a unifying moderator for listeners' emotional response to microtiming in swing and funk.
– *Study C* used the data from both experiments in order to compare the effect of fixed microtiming displacements (from *Study A*) with scaled versions of the originally performed microtiming patterns (from *Study B*). It showed that fixed snare drum displacements irritated expert listeners more than the more flexible deviations occurring in the original performances. This provides some evidence that listeners' emotional response to microtiming deviations not only depends on the magnitude of the deviations, but also on the kind and origin of the microtiming patterns (fixed lab displacements vs. flexible performance microtiming).

## 1. Introduction

*Microtiming* in music denotes a wide range of time-related phenomena that arise as a consequence of conflicting concepts of musical time. Music theory describes the organization of time in metered Western popular or art music using a few discrete, rationally related time duration categories (Hasty, 1997; London, 2004). The canonical notation of rhythm in Western music epitomizes this principle: the relation between the duration of different rhythmic values can be expressed as small-integer ratios. Given a specific tempo, a sequence of notes of the same rhythmic value (e.g., quarter notes) is thought to be isochronous. And notes written on the same metric position (e.g., chords) are supposed to begin synchronously. Performed music, however, plays in continuous physical time where musical events may happen at any moment. In performance, the occurrence of musical events shows considerable temporal freedom, and time intervals between events are variable. This fundamental discrepancy between theoretical, fixed, discrete duration categories and the flexible nature of music performance in continuous time broadly defines the domain of microtiming in metered Western music.

Microtiming phenomena are very diverse and differ across musical contexts. Some manifestations of microtiming are relatively large-scale, can easily be detected by the untrained ear, and have become characteristic for certain musical styles. The asymmetric subdivision of the beat, for example, is a frequent feature of both jazz (for an overview on the swing ratio literature, see Friberg and Sundström, 2002; Dittmar et al., 2015; Camara, 2016) and baroque music (Moelants, 2011). Further, expressive timing (i.e., local tempo variations) is a phenomenon widely observed in the performance of Western art music. It has been associated with phrase structure, harmonic and melodic development, and it is understood to be a major source of expressiveness in Western art music (Clarke, 1982; Cook, 1987; Repp, 1992, 1997, 1998b; Beran and Mazzola, 2000; Hong, 2003; Sundberg, 2003; Cheng and Chew, 2008; Senn et al., 2009, 2012; Dodson, 2011; MacRitchie, 2011; Benadon and Zanette, 2015).

A multitude of musical or contextual factors may be associated with microtiming phenomena. Tempo, for example, has been shown to be related with microtiming magnitude: Friberg and Sundström (2002) found that the swing ratio (i.e., the duration ratio between the long first and the short second swing eighth note) is negatively correlated with tempo. Moelants (2011) observed a comparable relationship for the baroque *notes inégales*, and Repp (1995) reported a similar tendency for the execution of expressive timing in Nineteenth century piano music: faster tempo implied less microtiming (see also Fraisse, 1956; Repp et al., 2002).

Bengtsson (1974) showed that the first quarter note of a Viennese Waltz measure tends to be longer than the second and third quarter notes. This can be associated with the dance movements: the basic Waltz step pattern begins with a long step, followed by two shorter ones. The microtemporally adapted beat durations help dancers to map their steps to the music more easily. Hence, the fact that the Waltz is used for dancing has shaped microtiming properties of the music.

Finally, the use of *tempo rubato* in the performance of Western art music piano repertoire has changed through performance history. Pianists of the early Twentieth century used ample *rubato*, but it fell out of favor during the second half of the century (Philip, 2004). Influential performers played a role (e.g., Glenn Gould and his recordings of works by J. S. Bach) but also new performance paradigms (such as the historically informed performance movement). In this case, the taste and aesthetics of the performers and of their audiences were contextual factors that modified the use of a microtiming-based performance technique like *tempo rubato*.

Within the same context, microtiming patterns might be so stable that they can even be cast into fixed performance rule systems (Beran and Mazzola, 2000; Friberg et al., 2006). Listeners' familiarity with such stable patterns may also affect their sensitivity to microtiming. Repp exemplified this in a striking way: he showed that listeners of Chopin's Etude op. 10, No. 3, had “obligatory expectations” with respect to the microtiming pattern, depending on phrase structure and an implicit rule system (Repp, 1998a).

The pioneers of technology-based timing analysis, Ingmar Bengtsson and Alf Gabrielsson, discussed the question to what extent small-scale microtiming phenomena are a meaningful part of the performance or just motor noise resulting from humans' limited capacity to realize a “quantized” music performance, i.e., a performance with perfect mechanical regularity (Gabrielsson, 1974; Bengtsson and Gabrielsson, 1977, 1980, see also Rasch, 1988). A large body of research on sensori-motor synchronization (for an overview of the tapping literature, see Repp, 2005; Repp and Su, 2013) studied the production side of this question, namely humans' timing precision when synchronizing body motion with acoustic stimuli. Some of these studies addressed the precision of expert drummers' performance (Fischinger, 2009; Fujii et al., 2011; Kilchenmann and Senn, 2011) and discussed methods to distinguish between unsystematic, random timing variations that are merely motor noise, and systematic, recurring variations that may be characteristic for a style and relevant for the effect of the music (Hellmer and Madison, 2015).

The perception of microtiming has also been studied: a substantial body of work discusses the precision of human timing discrimination with respect to auditory stimuli (Michon, 1964; Fraisse, 1967; Nordmark, 1968; Abel, 1972; Lunney, 1974; Halpern and Darwin, 1982; Hibi, 1983; Nakajima, 1987; Hirsh et al., 1990; Nakajima et al., 1992; Drake and Botte, 1993; Hoopen et al., 1995; Sasaki et al., 1998; Ehrlé and Samson, 2005; Thomas, 2007). In their influential study, Friberg and Sundberg (1995) found that the *Just Noticeable Difference* (*JND*) for microtiming deviations in a monotonic, isochronous sequence was approximately constant at an absolute value of 6 ms for inter-onset-intervals (IOIs) smaller than 240 ms. The *JND* remained stable at roughly 2.5% of the IOI when the interval was in the range 240–1,000 ms. Hence, above the 6 ms threshold, listeners' sensitivity to microtiming deviations appears to follow Weber-Fechner's law. *JND* research investigated listeners' time discrimination in a laboratory setting that is optimized for the detection of minimal deviations. In everyday listening situations, however, performed music is usually more complex than the lab stimuli, and listeners' threshold for detecting microtiming deviations in a regular listening context is likely to differ from the *JND*'s.

In recent years, the impact of microtiming on the groove phenomenon has been a major research topic. Relevant papers in music psychology define groove as an enjoyable inner urge to synchronize body movement with the beat of the music (Janata et al., 2012; Witek et al., 2014). This shifts the focus from the perceptual detectability or noticeability of microtiming to bodily entrainment and emotional responses.

The hypothesis that microtiming is essential for triggering groove originated in Charles Keil's *Theory of Participatory Discrepancies* or *PD Theory* (Keil, 1987, 1995, 2010). It has considerable support among musicians (Berliner, 1994; Monson, 1996; Doffman, 2008). The empirical evidence concerning the relevance of microtiming for groove, however, has been inconclusive so far: some studies found no evidence that microtiming influences groove (Butterfield, 2010; Madison et al., 2011; Madison and Sioros, 2014), others found that microtiming is detrimental to groove (Davies et al., 2013; Frühauf et al., 2013; Matsushita and Nomura, 2016); yet others found that microtiming patterns arising in competent performance do not affect groove negatively, but if the patterns are exaggerated in magnitude, the groove experience decreases (Kilchenmann and Senn, 2015; Senn et al., 2016). Finally, Hofmann et al. (2017) suggested that listeners preferred tightened microtiming patterns to the microtiming magnitude of the originally performed music.

How can we explain the discrepancies between these empirical results? Previous research has primarily focused on the magnitude of microtiming deviations. The patterning of the microtiming deviations have received little attention up to now. This paper presents three small studies that aim at exemplifying how patterning aspects may impact listeners' emotional responses to microtiming.

– *Study A* investigates to what extent the distribution of microtiming deviations across instrumental parts affects listeners' emotional responses. Previous studies have presented two methods for introducing fixed timing displacements into otherwise quantized musical stimuli: one method is to shift the entire parts played by different instruments against each other, for example a bass track against the corresponding drum track (as used in Butterfield, 2010; Matsushita and Nomura, 2016). The other method consists in displacing events against each other that are played by the same instrument: Frühauf et al. (2013), for example, displaced the snare drum or bass drum events relative to the rest of the drum track. The first method leaves the relationships within an instrumental part intact, whereas the second method introduces a temporal disturbance into a part. In *Study A*, both manipulation methods are applied to duo bass and drums recordings that have first been presented in Kilchenmann and Senn (2015) and used again in Senn et al. (2016). This allows for the comparison of the two manipulation methods' effects on the basis of the same musical examples, and it allows to verify the results of Butterfield (2010) and Frühauf et al. (2013).
– *Study B* uses data from an earlier experiment (Senn et al., 2016) to investigate how the magnitude of microtiming deviations can be quantified in a way that is meaningful and potentially uniform across different musical contexts. In Senn et al. (2016), the originally performed microtiming patterns of swing and funk duo performances (bass/drums) were down- or up-scaled by fixed percentages. The original swing performance showed greater average microtiming magnitudes in milliseconds than the funk performance. These differences in magnitude were accentuated when they were upscaled. But surprisingly, listeners' responses were indistinguishable across the two styles regardless of the scaling. This suggested that listeners expected to hear larger microtiming deviations in swing than in funk. The re-analysis of the data aims at studying how well listener responses align across styles when they are modeled as a function of three different measures for microtiming magnitude: the *Standard Timing Deviation* measures the mean deviations in milliseconds. The *Tempo-adjusted Standard Timing Deviation* accounts for tempo differences, treats tempo as a potential moderator variable, and measures microtiming magnitude as a proportion of the beat. The *Density-adjusted Standard Timing Deviation* considers rhythmic density as a moderator and measures microtiming as a proportion of the mean IOI of the music.
– *Study C*, finally, uses the data from *Studies A* and *B* to compare the effects of different microtiming patterns on listeners' emotional response. It asks specifically, whether listeners react more sensitively to the lab-generated, fixed microtiming displacements of *Study A* in comparison to the microtiming patterns that arose in the original performance, as used in *Study B*.

## 2. Study A: Microtiming between and within sound sources

*Study A* compares the groove-related effects of two laboratory-generated methods for systematically introducing fixed microtiming deviations into quantized music. The two manipulations affect entire instrumental parts or voices (i.e., layers of the musical fabric that are characterized by specific timbres) differently: the *Shift* manipulation displaces parts that are played by different musicians against each other by a certain time offset (between musicians). The *Disturbance* manipulation displaces voices against each other that are played by the same musician (within musician).

Butterfield (2010) used the *Shift* method: he worked with standard-practice jazz rhythm section examples (bass and drums). He displaced the drum tracks and the bass track relative to each other by either anticipating the bass onsets by 10, 20, or 30 ms relative to the drums (bass lead) or by conversely anticipating the drum onsets by 10, 20, or 30 ms (drums lead) relative to the bass. Butterfield found that this kind of manipulation had little effect on listeners.

Frühauf et al. (2013) took the *Disturbance* approach: they used a generic rock/pop drum pattern (eighth notes on the hi-hat, downbeats on the bass drum, and backbeats on the snare drum) and manipulated one layer within the drum track. They displaced either all bass drum or all snare drum sounds uniformly by −25, −15, 0, 15, or 25 ms, while keeping the remaining instruments in place. Frühauf et al. found a dose-response effect of the displacements on listeners' groove ratings: the perfectly quantized stimulus (0 ms displacement) obtained the highest groove ratings. Ratings decreased with larger displacements in either direction (early or late) and for either of the instruments. However, this effect was stronger when the snare drum was displaced instead of the bass drum, and when the displaced event onsets were early instead of late.

Matsushita and Nomura (2016) proceeded according to the *Shift* method. They used the same standard rock/pop drum pattern as Frühauf et al. (2013), but combined it with repeated eighth notes on a bass guitar. Compared to Butterfield (2010), they increased the magnitude of the time shift considerably: the bass voice was displaced by −62.50, −46.88, −31.25, 0, +31.25, +46.88, and +62.50 ms (negative numbers refer to bass lead, positive numbers to bass lag). They found that groove ratings were high for well synchronized stimuli, and declined with larger displacements in both bass lead and bass lag direction; these results were similar to those reported by Frühauf et al. (2013).

The goal of *Study A* is to replicate the main results of Butterfield (2010) and Frühauf et al. (2013) by applying both displacement methods to the swing and funk examples from Kilchenmann and Senn (2015). In particular, we try to verify, whether introducing a *Disturbance* microtiming pattern by displacing the snare drum layer against the other layers (the remaining drum tracks and the bass) affects the groove experience negatively (comparable to the effects found by Frühauf et al., 2013), and whether introducing a time *Shift* microtiming pattern between bass and drums has little or no effects on listeners (comparable to Butterfield, 2010).

### 2.1. Materials and methods

#### 2.1.1. Stimuli

The stimuli for both experiments reported in the present paper were derived from two recorded studio performances played by bassist Wolfgang Zwiauer and drummer Dominik Burkhalter, two internationally renowned performers on their respective instruments, professors at the Lucerne University of Applied Sciences and Arts, and musical collaborators for many years. The studio session was organized by the researchers, and its only purpose was to create two recordings from which experimental stimuli would subsequently be derived. In one of the two recordings, the musicians extemporized a funk pattern over an eight-bar vamp of their own invention, at a tempo of 100 bpm. In the other recording, the musicians improvised a swing pattern over a twelve-bar harmonic model at a tempo of 150 bpm. The chosen tempi are typical medium tempi within their respective genre contexts. Each original recording had a duration of approximately three minutes. The drummer played an acoustic drum set. The bassist played an electric bass for the funk recording and an acoustic bass guitar for the swing recording.

The musicians wore headphones during the studio performances. In the monitor mix, they heard the performed music and a metronome click as a common beat reference. The musicians sat acoustically separated in different recording booths and had direct visual contact through a glass panel. From previous studio work, the musicians were used to hearing a metronome click while performing, and they declared to be comfortable with this setup. After the recording session, the musicians indicated passages in each recording that they considered to have the best groove. From these passages, the researchers subsequently chose one iteration of the swing and funk patterns as a basis for the timing manipulations; the musicians agreed with this choice. These selections of 20 s duration have been used in previous studies (Kilchenmann and Senn, 2015; Senn et al., 2016) for the creation of experimental stimuli.

**Figure 1:**
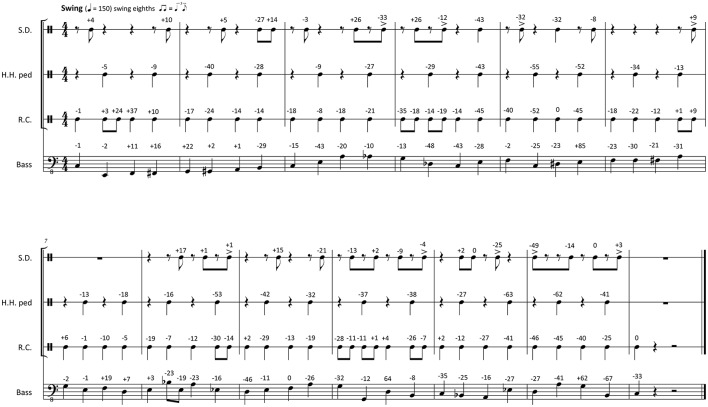
Transcription of the recorded 12-bar *Swing* pattern used as a basis for the timing manipulations. Originally performed timing deviations from metronomic time are indicated in milliseconds (negative numbers, event is ahead of the metronome; positive number, event sounds later than the metronome); S.D., snare drum; H.H. ped, foot-operated hi-hat cymbal; R.C., ride cymbal.

Transcriptions of the selected passages can be studied in Figure 1, 2. These transcriptions were made after the recording session, and they are of purely descriptive nature (Seeger, 1958). The musicians verified the accuracy and ecological validity of the transcriptions. The originally performed onset times were measured using the LARA analysis software^1^. An equidistant metronomic grid was derived from the click track, which defined a quantized onset time for each event of the performance. For swing, the mean swing ratio of the recording was used to determine the offbeat eighth notes grid positions. The mean swing ratio was 2.66, which is typical for jazz at 150 bpm (Friberg and Sundström, 2002; Dittmar et al., 2015). The transcriptions show the timing differences between the performed events and the corresponding position on the metronomic grid in milliseconds (negative numbers indicate that the performed onset was early; positive numbers indicate late onsets).

**Figure 2:**
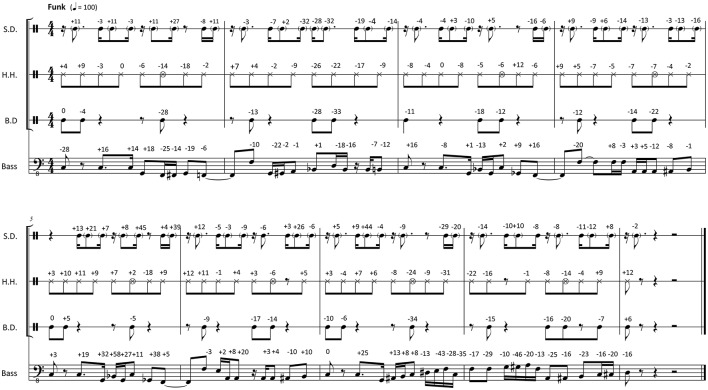
Transcription of the recorded 8-bar *Funk* pattern used as a basis for the timing manipulations. Originally performed timing deviations from metronomic time are indicated in milliseconds (negative numbers, event is ahead of the metronome; positive number, event sounds later than the metronome); S.D., snare drum; H.H., hi-hat cymbal; B.D., bass drum.

The drum audio track was replaced by an accurate reconstruction using the *Massey Drum Replacement Tool* (version 3.9) and samples from the *Toontrack Superior Custom and Vintage* library (version 2.3.1). This replacement was necessary in order to avoid creating acoustic artifacts when manipulating the timing of the events: such artifacts are a consequence of poor source separation when recording the different instruments of the drum set with several microphones. After replacement, the researchers obtained highly accurate replicas of the two selected passages that could be subjected to timing manipulations. No replacement was necessary for manipulating the timing of the bass line: *Avid Pro Tools*' (version 10.0.0) time stretching function was applied to the originally recorded bass audio track in order to create the bass tracks of the experimental stimuli. For *Study A*, all events were first adjusted to coincide exactly with the metronomic grid. Hence the subsequent *Shift* and *Disturbance* timing manipulations departed from perfectly quantized stimuli. (The original performance microtiming patterns will be used in *Study B*.)

In order to emulate the time *Shift* between the two instruments, as used by Butterfield (2010), a series of 14 experimental stimuli was created, in which the entire bass voice was displaced relative to the drums track by −24, −16, −8, 0, +8, +16, or +24 ms (seven stimuli for each style). The time shift manipulation introduced a time offset between the two instrumental parts, but the perfect quantization of the events within the bass and drums tracks was left intact.

In order to introduce *Disturbance* into the music (similar to Frühauf et al., 2013), 14 stimuli with timing irregularities were created by displacing the snare drum events by −24, −16, −8, 0, +8, +16, or +24 ms, while all the other events remained at their exact quantized positions (i.e., the bass and all other instruments of the drum set).

In summary, 28 experimental stimuli were derived from recordings in two *Styles* (*Funk* and *Swing*) by applying two microtiming manipulation methods (*Shift* and *Disturbance*) and seven different *Displacement Magnitudes* (−24, −16, −8, 0, +8, +16, or +24 ms) for each method. The stimuli for *Study A* can be downloaded from the Supplemental Material section of this article.

#### 2.1.2. Participants

A total of 160 participants took part in the listening experiment, 75 female and 85 male. One hundred and forty participants were students of Lucerne University of Applied Sciences and Arts, 18 were enrolled at Lucerne University, the remaining 2 participants were not affiliated with any University. Eighty two participants were considered to be music *Experts* because they were either enrolled in a program to become professional musicians or music teachers, or they had previously completed such a program. The remaining 78 participants did not meet either of these criteria and were thus considered to be musical *Non-Experts*. The participants were predominantly young adults ranging from 18 to 47 years ($\bar{x}=24$, *s* = 4.5). They were recruited via emails, class visits, and personal contacts. Since the questionnaires were in German, only fluent German speakers were recruited.

#### 2.1.3. Procedure

The experiment was carried out in a quiet office room at Lucerne School of Music. Participants were seated at a desk; stimuli were played from a personal computer (running the *Neurobs Presentation Software*, version 16, on *Windows 7*) through a *Presonus Firebox* audio interface and *AKG Mk II* headphones. The participants took the test one at a time. Written instructions informed each participant about the procedure of the experiment and about the possibility of aborting the experiment at any time. Participants adapted the size of the headphones and adjusted the playback volume to a comfortable level.

The experiment started with practice trials. Each participant assessed four test examples, and could ask questions to the experimenter if any aspect of the rating procedure or the navigation was unclear. When the participant declared to be familiar with the procedure, the investigator left the room, while the participant listened to the experimental stimuli and rated them using an on-screen rating form. In order to avoid style preference effects due to direct comparison, each participant listened to the stimuli of only one randomly assigned *Style*. The sequence of the 14 stimuli (seven in the *Shift* series, and seven in the *Disturbance* series) was randomized to counteract the effects of fatigue and familiarity. Design and procedure of the experiment were approved by the Ethics Committee of the Canton of Lucerne.

#### 2.1.4. Psychometric measures

Participants rated the stimuli using the *Emotional Assessment of Groove* (*EAG*) questionnaire. The *EAG* was constructed by the authors and was designed to capture emotional responses connected to the groove experience. It was validated in a pre-study at Justus-Liebig-University Giessen and used in a previous study (Senn et al., 2016). The questionnaire collects listeners' feedback on three scales that measure the strength of emotional reactions connected to the experience of groove: *Entrainment* (four items, Cronbach's α = 0.89), which measures how much the music stimulates the urge for body movement in listeners; *Enjoyment* (five items, Cronbach's α = 0.88), which measures the pleasure a participant experiences while listening to a stimulus. A third scale captures the participants' experience of unnaturalness or *Irritation* (four items, Cronbach's α = 0.94) while listening. This scale inversely measures the experience of effortlessness or fluency that has been associated with groove (Janata et al., 2012). The mental experiences captured by the three scales comply with the four-dimensional definition of emotions by Cabanac (2002).

Additionally, the well-established pictorial *Self Assessment Manikin* (*SAM*) was used to measure the affective reactions of participants to each listening experience (Bradley and Lang, 1994; Backs et al., 2005). The *SAM* measures affective reactions on three scales: *Valence* (happy vs. unhappy), *Arousal* (quiet vs. excited), and *Dominance* (powerful vs. powerless). It has been previously used for measuring reactions to music (Gomez and Danuser, 2007; Senn et al., 2016).

#### 2.1.5. Statistical design

Quadratic regression models were fitted to the data in order to test for effects of the seven timing *Displacement Magnitudes* (−24, −16, −8, 0, +8, +16, +24 ms) on any of the *EAG* (*Entrainment, Enjoyment, Irritation*) or *SAM* (*Valence, Arousal, Dominance*) outcome variables. The analysis was carried out separately for each of the two timing manipulation methods (*Shift, Disturbance*). The inclusion of quadratic regression coefficients was based on previous results that parabola-shaped regression models appear to best approximate this kind of data (see Frühauf et al., 2013; Matsushita and Nomura, 2016), compared to first-order linear regression models. The between-subjects variables *Style* (*Funk, Swing*) and *Expertise* (*Experts, Non-Experts*) did not show any effect on the outcome variables, hence they will be omitted in the Results section of *Study A*.

The presentation of the 14 stimuli as one randomized sequence caused a subtle, but serious methodological problem: the two quantized stimuli with 0 ms deviation in both the *Shift* and the *Disturbance* series were identical. Consequently, the association of the ratings with a stimulus of either series is purely coincidental. In order to solve this problem, the responses to the quantized stimuli were discarded from the dataset prior to the analysis.

The overall significance probability level was set to α = 0.05. With two displacement timing manipulation methods and six dependent variables (three each for *EAG* and *SAM*), a total of twelve independent models was fitted to the data. Šidàk correction was applied for familywise error protection (Šidàk, 1967; Huberty and Morris, 1989). Results were considered to be significant, when the Šidàk-corrected significance probability did not exceed α_š_ = 0.004.

### 2.2. Results

Regression model coefficients are presented in Table 1. Student's *t*-test was used to assess whether the linear or quadratic coefficients are significantly different from zero (which indicates that the ratings were affected by the timing manipulations). No tests were carried out with respect to the constant coefficients (intercept), because the *EAG* and *SAM* measures are greater than zero by design.

**Table 1:** *Study A*: Regression models for the relationships between *Displacement Magnitude* and the *EAG* and *SAM* response variables, separated by *Timing Manipulation Method*.

**DV**	**Method**	**Coefficient**	**Estimate**	***SE***	***df***	***t***	***p***
*Entrainment*	*Shift*	Constant	3.183	0.084			
		Linear	−0.084	1.305	798	−0.064	0.949
		Quadratic	−159.190	106.786	798	−1.491	0.136
	*Disturbance*	Constant	3.224	0.082			
		Linear	−0.063	1.418	798	−0.044	0.965
		Quadratic	−344.911	116.040	798	−2.972	0.003^*^
*Enjoyment*	*Shift*	Constant	3.050	0.071			
		Linear	0.714	1.355	798	0.527	0.598
		Quadratic	−186.344	110.872	798	−1.681	0.093
	*Disturbance*	Constant	3.083	0.073			
		Linear	0.089	1.383	798	0.065	0.949
		Quadratic	−368.503	113.143	798	−3.257	0.001^*^
*Irritation*	*Shift*	Constant	1.929	0.070			
		Linear	1.004	1.451	798	0.692	0.489
		Quadratic	176.877	118.699	798	1.490	0.137
	*Disturbance*	Constant	1.894	0.074			
		Linear	−0.991	1.583	798	−0.626	0.532
		Quadratic	582.450	129.503	798	4.498	< 0.001^*^
*Valence*	*Shift*	Constant	6.359	0.131			
		Linear	−1.981	2.524	798	−0.785	0.433
		Quadratic	−26.407	206.549	798	−0.128	0.898
	*Disturbance*	Constant	6.447	0.138			
		Linear	4.241	2.702	798	1.570	0.117
		Quadratic	−625.797	221.085	798	−2.831	0.005
*Arousal*	*Shift*	Constant	4.241	0.135			
		Linear	−1.032	2.430	798	−0.425	0.671
		Quadratic	−64.274	198.848	798	−0.323	0.747
	*Disturbance*	Constant	4.252	0.135			
		Linear	3.237	2.539	798	1.275	0.203
		Quadratic	−145.488	207.732	798	−0.700	0.484
*Dominance*	*Shift*	Constant	4.560	0.111			
		Linear	1.144	2.255	798	0.507	0.612
		Quadratic	133.032	184.536	798	0.721	0.471
	*Disturbance*	Constant	4.637	0.113			
		Linear	1.367	2.255	798	0.606	0.545
		Quadratic	−188.835	184.555	798	−1.023	0.307

DV, dependent variable; SE, standard error of the estimate; df, degrees of freedom; t, t-value; p, significance probability;

* *p < 0.004*.

**Figure 3:**
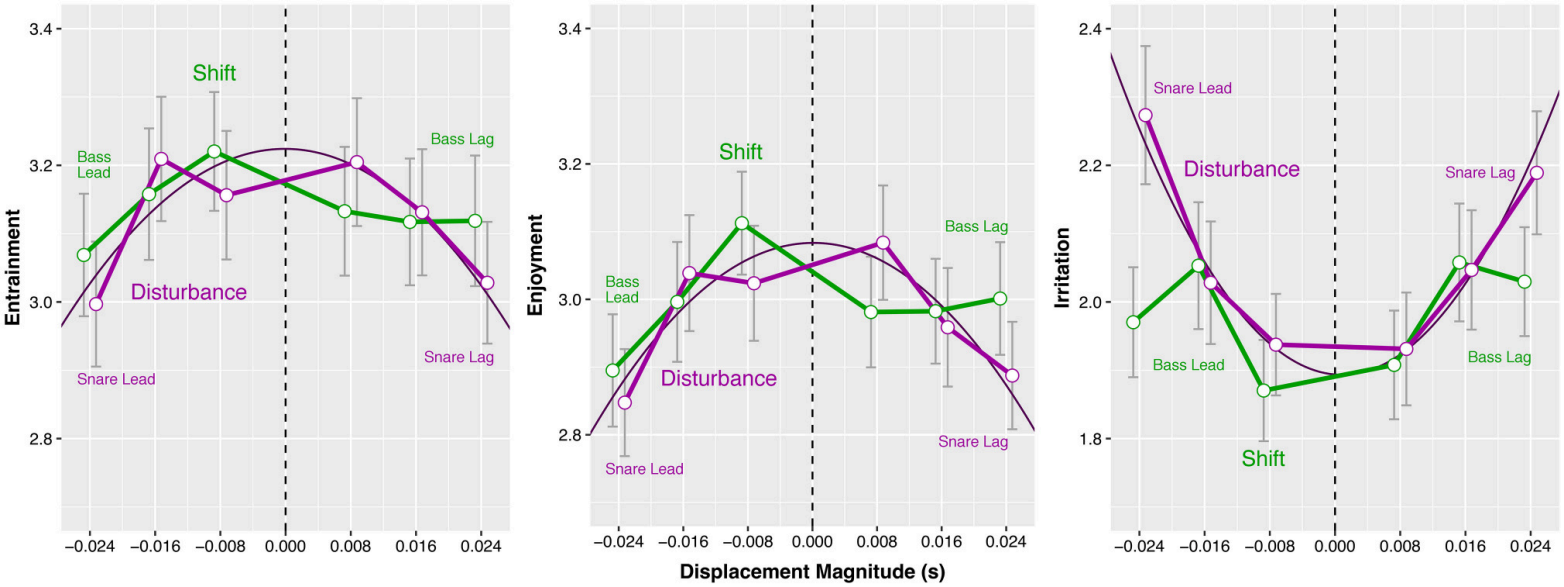
*Study A*: Effects of *Displacement Magnitude* (in seconds) on mean *Entrainment, Enjoyment*, and *Irritation* ratings, separated by microtiming manipulation method (*Shift* in green, *Disturbance* in purple). Error bars represent the standard error of the mean.

We observe that, for the *Disturbance* series (purple plots in Figure 3), the quadratic coefficients of the *EAG* scales (*Entrainment, Enjoyment*, and *Irritation*) differ significantly from zero. The corresponding linear coefficients are not significantly different from zero (this implies that the quadratic models are fairly symmetric about a *Displacement Magnitude* of 0 ms). Both, an increase of the displacements in negative (early) and positive (late) direction coincided with a decrease of the groove ratings. This manifests itself as a ∩-shaped plot in the case of *Entrainment* and *Enjoyment*, and as a ∪-shaped plot for *Irritation*. The quadratic models show that the *Disturbance* manipulation had significant effects on all three *EAG* response variables.

The largest effect was measured on *Irritation* (Cohen's *f*^2^ = 0.013), but this effect was very small according to Cohen's guidelines (Cohen, 1988). Effects on *Entrainment* (Cohen's *f*^2^ = 0.003) and *Enjoyment* (Cohen's *f*^2^ = 0.005) were significant, but even smaller. No significant effects were measured for the three *SAM* response variables. The effect of *Disturbance* on the *SAM*'s *Valence* scale (*p* = 0.005) slightly exceeded the Šidàk-corrected significance limit of α_š_ = 0.004; accordingly, it was not interpreted. The *Shift* manipulation (green plots in Figure 3) had no measurable effect on any of the response variables.

### 2.3. Discussion

The results from the *Disturbance* series (purple plots in Figure 3) confirm the central finding of Frühauf et al. (2013). In their study, groove ratings declined as one instrument of the drum set was displaced relative to the quantized pattern of the other instruments. Similar to the Frühauf et al. study, our data shows a dose-response relationship: larger absolute displacements of the snare drum events in negative (early) or positive (late) direction are associated with lower groove ratings. In Senn et al. (2016), we argued that the response measured by Frühauf et al. (2013) might be explained using results from research on attention, which showed that irregular signals perceived against a regular background can easily be detected (Scerbo et al., 1986; Bregman, 1999; Parasuraman, 2000; Helton et al., 2005; Dalton et al., 2007; Winkler et al., 2009). We hypothesize that timing manipulations as those used in Frühauf et al. (2013) or the *Disturbance* pattern of *Study A* can be interpreted from the attention point of view: the manipulations introduce irregularities into an otherwise perfectly quantized and regular drum track, and the groove ratings decline as the listeners become more aware of these irregularities.

The largest effect size found by Frühauf et al. (2013) (η^2^ = 0.71) exceeds the effect sizes measured in the present study substantially. We can only guess why a similar timing manipulation led to responses which are very different in scale. One obvious reason is that *Study A* lost statistical power when data was discarded due to the methodological problem outlined above.

Another explanation considers musical content: the two studies used different rhythmic patterns. Frühauf et al. used a simple, generic rock beat. The displaced elements (bass drum or snare drum) appear on down- or backbeat positions that are crucial for the establishment of meter, and hence they are relatively exposed. In the *Study A* stimuli, the snare drum is much more busy and varied than in the drum pattern used by Frühauf et al. (2013). The snare drum events in the *Study A* stimuli occur on many different metric positions: they may appear on downbeats, but also on more obscure offbeat positions. In the *Swing* stimuli, the snare drum has the function of a “comping” instrument, which means that its contribution is rhythmically irregular, syncopated and arguably less relevant for the definition of the beat than the clear downbeat/backbeat pattern found in the Frühauf et al. stimuli (this flexibility of the snare drum is similar to the rhythmic flexibility of the piano, the prototypical comping instrument in the common jazz rhythm section, see Hodson, 2007, p. 33). Further, many snare drum events in the *Study A* stimuli were ghost notes (i.e., played very softly). Hypothetically, ghost notes might be less noticed than notes that are played loud. Finally, the stimuli of the present study featured an additional bass line which adds even more complexity. In summary, we hypothesize that the timing displacements in this study's stimuli have less impact in comparison to the displacements used by Frühauf et al., because they are less apparent due to the generally greater musical complexity and the fact that many displaced elements appear on less important metric positions, compared to the stimuli used in Frühauf et al.

We did not find any effect of the *Shift* manipulation on any of the response variables. This non-result complies with the findings of Butterfield (2010) who observed that the displacement of the bass and drums tracks against each other were not noticed by listeners. The non-effect seems to be consistent across both studies, even though the tasks set to the listeners were slightly different: Butterfield (2010) confronted his listeners with a perceptual discrimination task; listeners were prompted to decide which of the two instruments (bass or drums) played earlier, had a leading role, or played more assertively compared to the other instrument. In contrast, our *Study A* collected listeners' emotional reponses only. For neither of these listening reactions, the *Shift* manipulations had any effect, at least within the range of displacement magnitudes used in the two studies.

This non-effect challenges the explanation borrowed from attention research presented above: if listeners perceive irregularities against a regular background and react with lower groove ratings, why do they not react to the bass displacement against the regular drum beat? After all, the bass and the snare drum each represent one prominent layer, voice, or auditory stream (Bregman, 1999) of the musical fabric. One potentially crucial aspect is that the bass and the entire drum set are considered to be individual sound sources that are played by different musicians and occupy different locations in space. In contrast, the snare drum occupies approximately the same location as the other instruments of the drum set. Temporal displacements between separate sound sources are part of our everyday listening experience. In dry air, at 20°C, the traveling speed of sound is approximately 343 ms^−1^; sound takes 0.0029 s (or 2.9 ms) to travel a distance of 1 m between a sound source and a listener. Listening in a physical environment is always linked to relative time delays between events created by different sound sources, placed at different locations. From this point of view, *Shift* manipulations are potentially decoded by the listeners as spatial information, not as an irregular foreground contrasting with a regular background. This might explain why the *Shift* manipulations did not have an effect on any of the response variables. Inverting the argument, we may claim that the *Disturbance* manipulation indeed did have an effect, because the snare drum and the other instruments of the drum set are assumed to be located at the same place. Hence, timing discrepancies cannot be traced back to different localizations of the sounds, instead they must originate in irregularities within the performance of one musician.

Contrasting with these results, Matsushita and Nomura (2016), in their first experiment, measured a significant effect of the time *Shift* between bass and drums. The effect might be explained by the sheer magnitude of the displacements in their experiment: at maximum asynchrony, the instruments' onsets were 62.50 ms apart, which is more than double the magnitude used in the other studies. These large displacements represent a delay time corresponding to a distance of 21.4 m. Such a large distance between musicians is not realistic in a duo performance context. So, in this extreme case, listeners' spatial interpretation of the time delays potentially breaks down, and they perceive the delays as poor synchronization.

To summarize, we used an argument about the (imagined) spatial arrangement of instruments and their players to claim that the *Shift* manipulations may be decoded by listeners as spatial information, which does not trigger an emotional response, whereas the *Disturbance* manipulations are potentially heard as imprecise playing of one of the performers, which leads to a negative emotional response.

## 3. Study B: In search of a context-independent measure for microtiming magnitude

In Senn et al. (2016) we reported that listeners' emotional responses were similar for swing and funk when the originally performed microtiming patterns were scaled by the same percentages. This was a surprising result, because the microtiming deviations (measured in milliseconds) of the swing performance were more largely spread than the deviations of the funk performance, and this difference was accentuated when the microtiming deviations were upscaled. Do listeners have an implicit knowledge which microtiming magnitudes are appropriate in each style? In *Study B*, instead of accepting such an essentialist explanation, we will try to identify moderating factors that bridge the differences, using the *EAG* response data from Senn et al. (2016).

Three different measures of microtiming magnitude will be defined: the *Standard Timing Deviation* (*STD*) estimates the mean microtiming deviation per note onset (a bass tone, a stroke on a drum etc.) in milliseconds using the *Root Mean Squared Error* method. The *Tempo-adjusted Standard Timing Deviation* measures mean deviation as a proportion of beat duration. And the *Density-adjusted Standard Timing Deviation* measures it as a proportion of the mean IOI between neighboring rhythmic events.

The two adjusted measures translate the core result of Friberg and Sundberg's (1995) experiment into the domain of more complex musical stimuli: for the monotonic, isochronous sequences of their experiment, a change of IOIs can equivalently be understood as a manipulation of tempo or of rhythmic density. For more complex musical objects, these two aspects need to be separated. A complex pattern might move at a slow tempo, but still be rhythmically dense, and vice versa.

The *Tempo-adjusted STD* implements tempo as a moderator variable (see Baron and Kenny, 1986; Cohen, 2003), which, in Western popular music, is represented by the periodicity of the beat. In contrast, the *Density-adjusted STD* considers how frequently a listener obtains any kind of rhythmic information. The two measures lead to different values when applied to this study's swing and funk stimuli: the swing example has higher tempo (150 bpm) than the funk example (100 bpm), hence the *Tempo-adjusted STD* augments the microtiming magnitude of the swing example relative to the funk example. But the rhythmic density of the swing example is smaller than the density of the funk example, because the smallest subdivision in swing are eighth notes, whereas the funk example has an underlying sixteenth note pulse. Hence *Density-adjusted STD* will accentuate the microtiming magnitude of the funk example relative to the swing example.

In a second step, we will analyse listeners' emotional responses to the swing and funk stimuli as a function of the three different microtiming measures. If any of the measures successfully aligns listeners' responses to microtiming in both the swing or the funk contexts, this measure potentially can be used to uniformly quantify microtiming across different musical situations, and the adjusting factor can be understood as a unifying moderator variable.

### 3.1. Three versions of the *Standard Timing Deviation*

The three methods for calculating summary timing deviation measures are differently scaled variants of the *Root Mean Squared Error* or *RMSE*, which is widely used for estimating the spread of a stochastic variable around an expected value.

The simple *Standard Timing Deviation* (*STD*) is measured in seconds, and it is calculated as follows:

(1) $$STD=\sqrt{\frac{\text{1}}{n}\sum_{i=1}^{n}{\left(t_{i}-{\hat{t}}_{i}\right)}^{2}},$$

where *n* is the number of note onsets in the musical passage, *t*_*i*_ is the exact time of the *i*th note onset, and ${\hat{t}}_{i}$ is the corresponding quantized time point on the metronomic grid (or expected onset time). For the 20 s swing passage that is presented in Figure 1 (with the originally performed microtiming deviations in milliseconds given as numeric values next to each note), we calculate an *STD* value of 0.0272 s (27.2 ms). For the funk passage showed in Figure 2 we calculate a smaller value of 0.0157 s (15.7 ms).

The *Tempo-adjusted Standard Timing Deviation* was already introduced in Senn et al. (2016). Based on the findings of Friberg and Sundberg (1995) and Ehrlé and Samson (2005), we hypothesized that listeners would be more sensitive to microtiming deviations at higher tempi than at lower tempi. The *Tempo-adjusted STD* is measured as a proportion of the beat, and it is calculated as:

(2) $$Tempo\text{-}adjusted\;STD\text{=}\frac{\text{bpm}}{\text{60}}\sqrt{\frac{\text{1}}{n}\sum_{i=1}^{n}{\left(t_{i}-{\hat{t}}_{i}\right)}^{2}},$$

where bpm is the tempo of the music in beats per minute, and all other variables are the same as above. A comparison of Equations (1) and (2) shows that the tempo-adjustment is a scaling of the simple *Standard Timing Deviation* by the constant factor bpm/60, which represents the number of beats per second. For the swing passage with the originally performed microtiming, the *Tempo-adjusted STD* is 0.0681 (6.81% of the duration of a beat), whereas for the original funk clip, we obtain a value of 0.0262 (2.62% of the beat).

Before defining the *Density-adjusted Standard Timing Deviation*, we first define rhythmic density ρ_*R*_ as follows:

(3) $$\rho_{R}=\frac{E-1}{{\hat{t}}_{E}-{\hat{t}}_{1}}=\frac{1}{\bar{\text{IOI}}},$$

where *E* is the number of distinct events (a distinct event is a metronomic grid position on which at least one note onset occurs). Further, ${\hat{t}}_{1}$ and ${\hat{t}}_{E}$ are the expected times of the first and the last event, respectively. Rhythmic density ρ_*R*_ measures the mean number of distinct events per second, and it is the reciprocal of the mean inter-onset-interval ($\bar{\text{IOI}}$). The *Density-adjusted Standard Timing Deviation* is then calculated as

(4) $$Density\text{-}adjusted\;STD=\rho_{R}\sqrt{\frac{\text{1}}{n}\sum_{i=1}^{n}{\left(t_{i}-{\hat{t}}_{i}\right)}^{2}}$$

and it measures timing deviation as a proportion of $\bar{\text{IOI}}$. The swing passage with originally performed microtiming shows a *Density-adjusted STD* value of 0.1064 (or 10.64% of the $\bar{\text{IOI}}$), whereas the original funk performance has a value 0.0911 (or 9.11% of the mean inter-onset-interval).

### 3.2. Materials and methods

#### 3.2.1. Stimuli and timing manipulations

The stimuli for *Study B* were derived from the same two swing and funk recordings that were used in *Study A*. The timing was manipulated by scaling the originally performed microtiming deviations (see transcriptions and microtiming deviations given in Figure 1, 2) relative to the metronomic grid. The scaling had eleven different levels: −100, −80, −60, −40, −20, 0, +20, +40, +60, +80, +100%. On the 0% level, the microtiming deviations from the metronomic grid were exactly as in the originally recorded performance. The +100% level upscaled the original deviation of every note to double magnitude. And on the −100% level, the deviation of every note was downscaled so that all events occurred on the metronomic grid, and the music was perfectly quantized (with the swing example showing an eighth-note swing ratio of 2.66). The same procedure was applied to the examples from both styles, swing and funk, based on the microtiming deviations occurring in the original performance. The upscaling exaggerated the microtiming profiles of the original performances, whereas the downscaling flattened these profiles (for more details on the timing manipulations, see Kilchenmann and Senn, 2015; Senn et al., 2016). The stimuli for *Study B* can be downloaded from the Supplemental Material section of Kilchenmann and Senn (2015).

#### 3.2.2. Participants, procedure, psychometric measures, and statistical design

A total of *n* = 160 persons participated in the experiment, which was carried out at the Lucerne University of Applied Sciences and Arts. Seventy nine participants were considered to be music *Experts*, as they had either obtained a professional music degree (musicians or music educators), or were enrolled in programs to earn such a degree. The remaining 81 participants did not meet these criteria and were considered to be musical *Non-Experts*. Most of the participants were either affiliated with the Lucerne University of Applied Sciences and Arts or with Lucerne University. The sample consisted of 82 female and 78 male participants, their ages ranged from 18 to 47 years ($\bar{x}=24$, *s* = 4.3), and all participants were fluent German speakers. The majority of participants was recruited via emails and class visits, a few were invited through personal communication channels.

The experimental procedure of *Study B* was identical to the procedure employed in *Study A*; it was also approved by the Ethics Committee of the Canton of Lucerne. Participants performed the listening test alone in a quiet office. They followed on-screen instructions, listened to the stimuli through studio headphones and filled the questionnaires by mouseclick. Each participant heard all stimuli of one style, and the sequence of the presentation was randomized. The participants rated the stimuli using the *Emotional Assessment of Groove* (*EAG*) and *Self-Assessment Manikin* (*SAM*) questionnaires. Only the three *EAG* scales (*Entrainment, Enjoyment*, and *Irritation*) will be considered in the subsequent analyses. These three scales have been shown to record participants' emotional responses to microtiming manipulations quite consistently (see Senn et al., 2016 and *Study A*).

Statistical analyses were carried out separately for each of nine combinations of measurement method (*STD, Tempo-adjusted STD, Tempo-adjusted STD*) and outcome variable (*Entrainment, Enjoyment*, and *Irritation*). In each combination, regression models were fitted to the data, separated by *Style* (*Swing, Funk*), with the microtiming measurement as predictor, and listeners' emotional response as outcome variable. Quadratic models were used if they had a significantly better fit in both styles than alternative first-order linear models. Finally, model coefficients were compared across styles in order to determine whether the models were significantly different.

The overall significance level was set to α = 0.05. No correction was applied for familywise error protection, because Type I errors are no serious concern in *Study B*: the focus lies on non-significant test results, because they indicate that there is little evidence against the null hypothesis of no difference between listener reactions to *Swing* or *Funk* stimuli. Non-significant test results inform us that the respective microtiming measure predicts invariant listener responses across the two style contexts; hence it is potentially a generally applicable measure for microtiming, and the adjusting variable is a mediator.

### 3.3. Results

**Figure 4:**
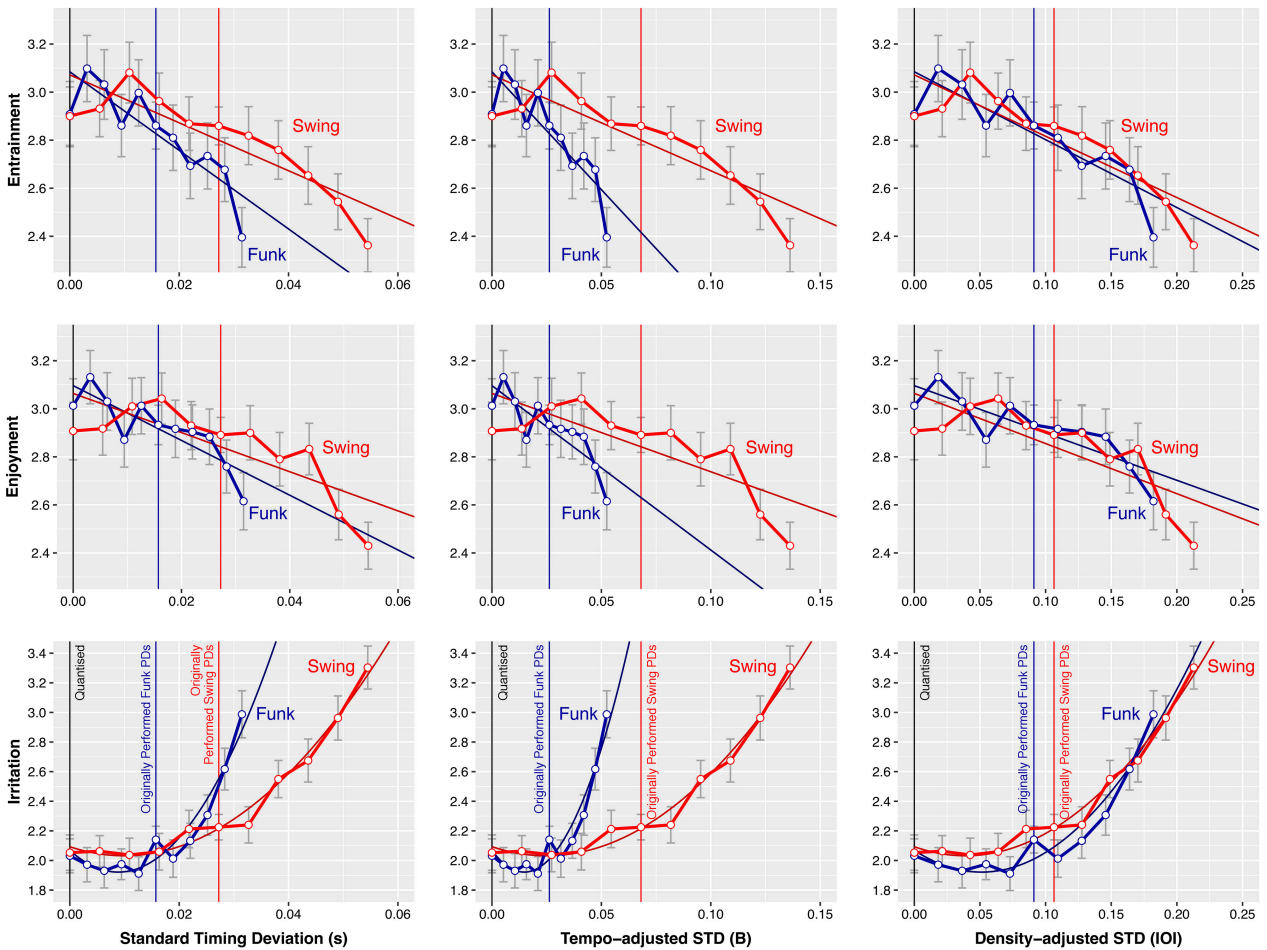
*Study B*: Mean *EAG* variables (*Entrainment, Enjoyment, Irritation*) as a function of three microtiming magnitude measures, separated by *Style* (*Swing, Funk*). The *Standard Timing Deviation* measures microtiming magnitude in seconds (s), the *Tempo-adjusted Standard Timing Deviation* measures it as a proportion of mean beat duration (B), and the *Density-adjusted Standard Timing Deviation* as a proportion of mean IOI. Error bars represent the standard error of the mean.

Figure 4 plots the three *EAG* response variables (*Entrainment, Enjoyment*, and *Irritation*) against the three summary microtiming measures (*STD, Tempo-adjusted STD*, and *Density-adjusted STD*) for each of the stimuli. Each row of diagrams refers to the same response variable, whereas each column of diagrams refers to the same timing deviation measure. Participants' responses to the eleven *Swing* stimuli are plotted in red; responses to the *Funk* stimuli are plotted in blue. In each of the nine plots, the stimuli with completely quantized timing in either style are shown to the far left and marked with a vertical black line. This position corresponds to a magnitude of zero on all three *Standard Timing Deviation* scales. The microtiming magnitudes of the original performances are marked with a red (*Swing*) or a blue (*Funk*) vertical line. These values differ for each *Style* and for each of the three *STD* measures. The responses to the stimuli with doubled microtiming deviations are furthest to the right in each plot.

As a general response pattern, *Entrainment* and *Enjoyment* ratings are high for small timing deviation values and decline with larger values; *Irritation* inverts this pattern (for a more detailed report, see Senn et al., 2016). We are now interested to study, to what extent response patterns differ or align across styles when the *EAG* ratings are represented as functions of either of the three timing deviation measures.

In order to compare the responses across styles for the different combinations of timing deviation measurements and *EAG* scales, either linear or quadratic regression models were fitted to the data. With respect to *Entrainment*, a quadratic model fits the *Swing* data better than a linear model [*F*_(1, 957)_ = 5.775, *p* = 0.016], but an additional quadratic term does not significantly improve the fit of the *Funk* data [*F*_(1, 945)_ = 2.123, *p* = 0.145]. Consequently only linear models were used to analyse the *Entrainment* data of both *Styles*. With respect to *Enjoyment*, the situation is similar: the quadratic term is significantly different from zero for *Swing* [*F*_(1, 957)_ = 8.690, *p* = 0.003], but not for *Funk* [*F*_(1, 945)_ = 1.060, *p* = 0.303], hence only linear models were fitted to the data. For the *Irritation* data, quadratic models have a significantly better fit than linear models for both *Styles* [*Swing*: *F*_(1, 957)_ = 19.791, *p* < 0.001; *Funk*: *F*_(1, 945)_ = 20.690, *p* < 0.001], hence quadratic models were used to compare the *Irritation* responses.

The model coefficients using the simple *Standard Timing Deviation* as predictor variable are presented in Table 2; the models are visualized as sloping straight lines (*Entrainment, Enjoyment*), or as parabolae (*Irritation*) in the left column of Figure 4. The linear models predict that *Entrainment* and *Enjoyment* are negatively related to *Standard Timing Deviation* for both *Swing* and *Funk*, as expected. As the *Standard Timing Deviation* increases, the *Entrainment* and *Enjoyment* ratings decline, indicating that the groove experience deteriorates with higher *Standard Timing Deviation*. For *Irritation*, the quadratic term was significantly greater than zero with respect to both the *Swing* and *Funk* datasets. The models predict that listeners' *Irritation* grows increasingly with higher values of *Standard Timing Deviation*.

**Table 2:** Regression models for the relationship between *Standard Timing Deviation* and *Entrainment, Enjoyment*, and *Irritation* for *Swing, Funk*, and the difference between *Swing* and *Funk*.

**Standard Timing Deviation**							
**DV**	**Style**	**Coefficient**	**Estimate**	***SE***	***df***	***t***	***p***
*Entrainment*	*Swing*	Constant	3.072	0.065			
		Linear	−9.974	2.050	958	−4.866	< 0.001^*^
	*Funk*	Constant	3.085	0.076			
		Linear	−16.354	4.108	946	−3.981	< 0.001^*^
	*Swing* – *Funk*	Constant	−0.013	0.153	205	−0.084	0.933
		Linear	6.380	2.813	1,747	2.268	0.023^*^
*Enjoyment*	*Swing*	Constant	3.064	0.060			
		Linear	−8.150	1.893	958	−4.306	< 0.001^*^
	*Funk*	Constant	3.096	0.064			
		Linear	−11.401	3.484	946	−3.272	0.001^*^
	*Swing* – *Funk*	Constant	−0.032	0.124	247	0.260	0.795
		Linear	3.250	2.921	1,747	1.113	0.266
*Irritation*	*Swing*	Constant	2.093	0.096			
		Linear	−12.289	7.872	957	−1.561	0.119
		Quadratic	616.639	138.610	957	4.449	< 0.001^*^
	*Funk*	Constant	2.068	0.095			
		Linear	−33.153	13.493	945	−2.457	0.014^*^
		Quadratic	1871.108	411.358	945	4.549	< 0.001^*^
	*Swing* – *Funk*	Constant	0.025	0.151	520	0.165	0.869
		Linear	20.864	12.940	1,745	1.612	0.107
		Quadratic	−1254.469	360.500	1,745	−3.480	< 0.001^*^

DV, dependent variable; SE, standard error of the estimate; df, degrees of freedom; t, t-value; p, significance probability;

* *p < 0.05*.

Comparative tests for the difference between *Swing* and *Funk* models are also presented in Table 2 (*Swing* – *Funk*). For *Entrainment*, the slope of the *Funk* linear model was significantly steeper than the slope of the *Swing* model. For *Enjoyment*, this difference was only nominal. For *Irritation* the quadratic term of the *Funk* model was significantly greater than the equivalent term of the *Swing* model. This means that the simple *Standard Timing Deviation* is sensitive to the style context: the models predict that listeners react more strongly to microtiming in *Funk* compared to *Swing*, if it is measured using the *Standard Timing Deviation*.

Table 3 presents a similar analysis, but this time using the *Tempo-adjusted Standard Timing Deviation* as a predictor variable. This analysis corresponds to the plots in the middle column of Figure 4. We observe that the *Swing* and *Funk* regression models are significantly different from each other for all three *EAG* response variables (*Entrainment, Enjoyment, Irritation*), hence the *Tempo-adjusted Standard Timing Deviation* is also sensitive to the style context.

**Table 3:** Regression models for the relationship between *Tempo-Adjusted STD* and *Entrainment, Enjoyment*, and *Irritation* for *Swing, Funk*, and the difference between *Swing* and *Funk*.

**Tempo-Adjusted Standard Timing Deviation**							
**DV**	**Style**	**Coefficient**	**Estimate**	***SE***	***df***	***t***	***p***
*Entrainment*	*Swing*	Constant	3.072	0.065			
		Linear	−3.990	0.820	958	−4.866	< 0.001^*^
	*Funk*	Constant	3.085	0.076			
		Linear	−9.812	2.465	946	−3.981	< 0.001^*^
	*Swing* – *Funk*	Constant	−0.013	0.153	205	−0.084	0.933
		Linear	5.823	1.567	1,747	3.715	< 0.001^*^
*Enjoyment*	*Swing*	Constant	3.064	0.060			
		Linear	−3.260	0.757	958	−4.306	< 0.001^*^
	*Funk*	Constant	3.096	0.064			
		Linear	−6.841	2.090	946	−3.272	0.001^*^
	*Swing* – *Funk*	Constant	−0.032	0.124	246	−0.260	0.795
		Linear	3.580	1.627	1,747	2.200	0.028^*^
*Irritation*	*Swing*	Constant	2.093	0.096			
		Linear	−4.916	3.149	957	−1.561	0.119
		Quadratic	98.662	22.178	957	4.449	< 0.001^*^
	*Funk*	Constant	2.068	0.095			
		Linear	−19.892	8.096	945	−2.457	0.014^*^
		Quadratic	673.600	148.089	945	4.549	< 0.001^*^
	*Swing* – *Funk*	Constant	0.025	0.151	520	0.165	0.869
		Linear	14.976	7.211	1,745	2.077	0.038^*^
		Quadratic	−574.937	124.515	1,745	−4.617	< 0.001^*^

DV, dependent variable; SE, standard error of the estimate; df, degrees of freedom; t, t-value; p, significance probability;

* *p < 0.05*.

Table 4 finally analyses the *EAG* responses with respect to the *Density-adjusted Standard Timing Deviation* as predictor variable. We observe that the differences between the models fitted to the *Swing* and *Funk* data are statistically insignificant. The models align quite closely, as can be seen in the rightmost column of Figure 4. This suggests that the *Density-adjusted Standard Timing Deviation* measure is relatively insensitive to the differences between the *Swing* and the *Funk* examples of *Study B*.

**Table 4:** Regression models for the relationship between *Density-Adjusted STD* and *Entrainment, Enjoyment*, and *Irritation* for *Swing, Funk*, and the difference between *Swing* and *Funk*.

**Density-Adjusted Standard Timing Deviation**							
**DV**	**Style**	**Coefficient**	**Estimate**	***SE***	***df***	***t***	***p***
*Entrainment*	*Swing*	Constant	3.072	0.065			
		Linear	−2.553	0.525	958	−4.866	< 0.001^*^
	*Funk*	Constant	3.085	0.076			
		Linear	−2.825	0.710	946	−3.981	< 0.001^*^
	*Swing* – *Funk*	Constant	−0.013	0.153	205	−0.084	0.933
		Linear	0.272	0.553	1,747	0.492	0.623
*Enjoyment*	*Swing*	Constant	3.064	0.060			
		Linear	−2.087	0.485	958	−4.306	< 0.001^*^
	*Funk*	Constant	3.096	0.064			
		Linear	−1.970	0.602	946	−3.272	0.001^*^
	*Swing* – *Funk*	Constant	−0.032	0.124	247	−0.260	0.795
		Linear	−0.117	0.575	1,747	−0.203	0.839
*Irritation*	*Swing*	Constant	2.093	0.096			
		Linear	−3.146	2.015	957	−1.561	0.119
		Quadratic	40.412	9.084	957	4.449	< 0.001^*^
	*Funk*	Constant	2.068	0.095			
		Linear	−5.728	2.331	945	−2.457	0.014^*^
		Quadratic	55.850	12.279	945	4.549	< 0.001^*^
	*Swing* – *Funk*	Constant	0.025	0.151	520	0.165	0.869
		Linear	2.582	2.546	1,745	1.014	0.311
		Quadratic	−15.438	12.633	1,745	−1.222	0.222

DV, dependent variable; SE, standard error of the estimate; df, degrees of freedom; t, t-value; p, significance probability;

* *p < 0.05*.

### 3.4. Discussion

If listener reactions were expressed as a function of the *Standard Timing Deviation* or of the *Tempo-adjusted Standard Timing Deviation*, the *Style* variable had a significant effect. In contrast, *Style* became irrelevant, when microtiming magnitudes were measured as *Density-adjusted Standard Timing Deviation*. The uniformity of responses across the two *Styles* suggests that the *Density-adjusted Standard Timing Deviation* is a more context-insensitive measure for the subjectively experienced microtiming magnitude, compared to the two other measures. Rhythmic density, potentially, is a moderating factor for listeners' emotional responses to microtiming.

Note that the originally performed microtiming magnitudes also aligned, when they were measured as *Density-adjusted STD*: in both the *Swing* and *Funk* examples, the originally performed mean microtiming magnitude amounted to approximately 10% of the music's mean IOI. The density-adjustment suggests that the musicians' performances show consistent microtiming across the two styles.

The main finding of *Study B* can be related to previous research:

1. The result agrees with the *perception*-oriented findings of Friberg and Sundberg (1995): similar to the just-noticeable differences, listeners' emotional responses to microtiming deviations appear to depend on rhythmic density or, reciprocally, on the mean time interval between subsequent events. Thus, they seem to obey Weber-Fechner's law: when time intervals between events are small, listeners are affected by small microtiming variations. But if intervals are large, it takes greater microtiming variations to have an effect on listeners.
2. The result is compatible with recent findings on *sensori-motor synchronization*: Madison (2014) found that tapping along with a musical stimulus was more accurate, when a higher number of metrical levels was present in the acoustic stimulus (for example eighth notes in addition to quarter notes). Adding metrical levels is equivalent to augmenting the rhythmic density without altering the tempo. We may assume that the precision of sensori-motor synchronization depends on tappers' perceptual sensitivity to timing discrepancies as studied by Friberg and Sundberg (1995) (only people who perceive that they are tapping off the beat are able to adapt their performance). Similarly, the emotional reactions to microtiming as measured by the *EAG* may also depend on listeners' sensitivity to timing discrepancies. The results of both Madison (2014) and *Study B* are presumably rooted in the same cognitive substrate that governs the *JND* of time perception (Friberg and Sundberg, 1995).
3. A further connection can be drawn to the *Theory of Attentional Dynamics* (Large and Jones, 1999; Jones et al., 2006) which discusses models of attention and expectation in the perception of periodically recurring events. This theory models the expected arrival time of an event as a bell-shaped probability distribution, which is updated every cycle in a quasi Bayesian way. The spread of the distribution (and hence the expected temporal variability of events) is thought to depend on the variability of past events and on the mean IOI between these events (see Large and Jones, 1999, p. 132). Hence, the *Theory of Attentional Dynamics* appears to associate the variables in a similar way as Friberg and Sundberg (1995) and the present study, but it adds a procedural perspective.

In summary, the idea that rhythmic density (but not tempo) might be a relevant moderating factor for listeners' emotional response to microtiming deviations seems to be compatible with theories on auditory perception, sensori-motor synchronization and dynamic attending. Nevertheless, our study's empirical evidence, based on two performances only, is too circumstantial to warrant a general claim. More research is necessary in order to study whether the scope of the result expands beyond these two particular recordings, beyond the playing of these two particular musicians at this particular day, and beyond the swing and funk styles. For the data at hand, however, the *Density-adjusted Standard Timing Deviation* appears to be our best guess of a metric that allows to compare listeners' response behavior across the different musical contexts.

## 4. Study C: Comparing the effect of lab- and performance-generated microtiming patterns on music experts and non-experts

The previous section presented some evidence that the *Density-adjusted Standard Timing Deviation* aligns listener responses to microtiming deviations across different musical contexts. This alleged invariance is not more than a claim at this point. But if confirmed by further research, it might provide a welcome common ground for the comparison of microtiming effects arising in different situations and contexts.

In *Study C*, we probe into the potential of such comparisons: we hypothesize that listeners respond differently to the invariant, lab-generated *Shift* and *Disturbance* patterns (*Study A*) compared to the variable microtiming pattern that arose in the original *Performances* (*Study B*). The rationale behind this claim is that we expect listeners to be more accustomed to *Performance* microtiming patterns than to *Shift* and *Disturbance* patterns. And we also hypothesize that *Expert* listeners react more strongly to microtiming deviations, compared to *Non-Expert* listeners, due to the *Experts*' training and refined auditory perception. In order to test these hypotheses, we will fit regression models to listeners' *EAG* ratings, using the *Density-adjusted STD* as a common predictor variable.

### 4.1. Method

No new listener responses were collected for *Study C*. Instead, the *EAG* groove ratings from *Studies A* and *B* were re-analyzed in order to test the hypotheses raised above. The *Density-adjusted STD* value was calculated for each stimulus, using Equation (4). These values are presented in Table 5.

**Table 5:** *Density-Adjusted STD* values for the stimuli of the *Shift, Disturbance* and *Performance* microtiming patterns.

**Pattern**	**Level**	**Density-adjusted STD**	
		***Funk***	***Swing***
*Shift* (A)	−8 ms	0.0215	0.0144
	−16 ms	0.0429	0.0289
	−24 ms	0.0664	0.0433
	+8 ms	0.0215	0.0144
	+16 ms	0.0429	0.0289
	+24 ms	0.0664	0.0433
*Disturbance* (A)	−8 ms	0.0209	0.0123
	−16 ms	0.0418	0.0246
	−24 ms	0.0627	0.0369
	+8 ms	0.0209	0.0123
	+16 ms	0.0418	0.0246
	+24 ms	0.0627	0.0369
*Performance* (B)	0%	0.0911	0.1064
	−20%	0.0729	0.0851
	−40%	0.0546	0.0639
	−60%	0.0364	0.0426
	−80%	0.0182	0.0213
	−100%	0.0000	0.0000

*The Shift series refers to displacements of the bass notes against the drum notes. The Disturbance series refers to displacements of the snare drum notes against the other notes. The manipulations of the Performance series indicate the percentage by which the displacements in the original performance were downscaled*.

We observe that, in the *Shift* and *Disturbance* series, negative and positive displacements of the same magnitude are projected onto the same *Density-adjusted STD* value for both the *Shift* and *Irregularity* series (this follows from the fact that negative and positive values are positive when squared). We also see that the originally performed *Swing* and *Funk* microtiming patterns (0%) show considerably larger *Density-adjusted STD*s than the largest values associated with the *Shift* and *Disturbance* series. This discrepancy is particularly striking for *Swing*. In order to compare data with an approximately similar range on the predictor variable, only listener responses to stimuli with originally performed (0%) and downscaled (−20, −40, −60, −80, −100%) microtiming were used for the regression analysis; the data corresponding to stimuli with exaggerated (upscaled) performance microtiming were not included.

The regression analyses test whether linear models fitted to the *Shift* and *Disturbance* listener response data (both from *Study A*) differ significantly from linear models fitted to the *Performance* data. The stimuli from the two *Styles* (*Funk, Swing*) will be analyzed separately. Similarly, the two *Expertise* groups (*Expert, Non-Expert*) will each be modeled on their own. With three *EAG* outcome variables (*Entrainment, Enjoyment, Irritation*) and four *Expertise*/*Style* combinations, twelve regression models will be fitted in parallel. The overall significance level is set to α = 0.05. There were twelve parallel comparisons, hence the Šidàk-corrected significance level was set to α_š_ = 0.004.

### 4.2. Results

**Figure 5:**
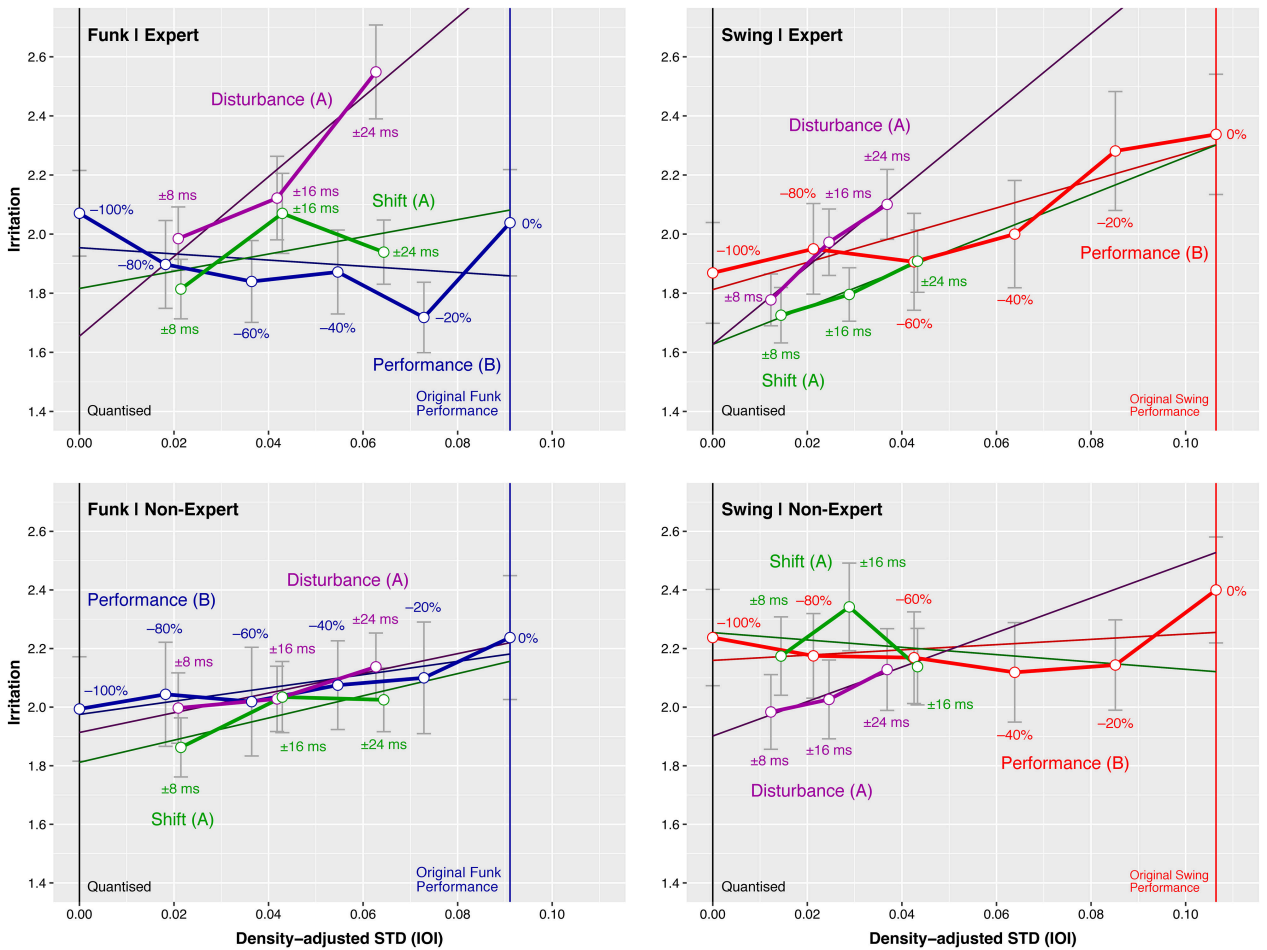
*Study C*: Mean *Irritation* as a function of the *Density-adjusted Standard Timing Deviation* (proportion of mean IOI) across different *Style*/*Expertise* combinations for the *Disturbance* (purple) and *Shift* (green) manipulation patterns and the *Performance* pattern (blue or red) scaled from quantized to original magnitude. Error bars represent the standard error of the mean.

Figure 5 plots the mean *Irritation* ratings against the *Density-adjusted STD* for the different stimuli series *Shift* (green), *Disturbance* (purple), and *Performance* (blue or red). The responses to the *Funk* stimuli with the different scalings of the original *Performance* microtiming pattern (from *Study B*) are shown in blue on the left, the *Swing* stimuli are presented in red on the right.

Test results for the *Irritation* response data are presented in Table 6. The *Shift* – *Performance* tests analyse whether the intercepts or the slopes of simple linear regression models fitted to the *Shift* series differ significantly from the models of the corresponding *Performance* series. Similarly, the *Disturbance*–*Performance* tests detect differences between the *Disturbance* and corresponding *Performance* models.

**Table 6:** Regression models for the relationship between *Density-adjusted STD* and *Irritation*.

**Style**	**Expertise**	**Pattern**	**Coefficient**	**Estimate**	***SE***	***df***	***t***	***p***
*Funk*	*Expert*	*Shift – Performance*	Constant	−0.356	0.205	702	−1.734	0.083
			Linear	3.958	3.780	650	1.047	0.295
		*Disturbance – Performance*	Constant	−0.517	0.205	702	−2.522	0.012
			Linear	14.536	3.855	650	3.771	< 0.001^*^
*Funk*	*Non-expert*	*Shift – Performance*	Constant	−0.086	0.179	709	−0.482	0.630
			Linear	1.522	3.079	642	0.494	0.621
		*Disturbance – Performance*	Constant	0.016	0.179	709	0.088	0.930
			Linear	1.099	3.142	642	0.350	0.727
*Swing*	*Expert*	*Shift – Performance*	Constant	−0.386	0.192	693	−2.014	0.044
			Linear	1.730	4.707	647	0.368	0.713
		*Disturbance – Performance*	Constant	−0.386	0.192	693	−2.014	0.044
			Linear	8.539	5.453	647	1.566	0.118
*Swing*	*Non-Expert*	*Shift – Performance*	Constant	0.093	0.208	676	0.448	0.654
			Linear	−2.151	5.061	622	−0.425	0.671
		*Disturbance – Performance*	Constant	−0.260	0.208	676	−1.250	0.212
			Linear	4.989	5.868	622	0.850	0.396

Study A timing displacement patterns (Shift, Disturbance) relative to the Performance patterns from Study B. SE, standard error of the estimate; df, degrees of freedom; t, t-value; p, significance probability;

* *p ≤ 0.004*.

Table 6 shows further that, for music *Experts* listening to *Funk*, the slope of the *Irritation* linear regression model fitted to the series with *Disturbance* pattern is significantly greater than the slope of the model fitted to the respective *Performance* pattern. We can state that music experts' *Irritation* increased significantly stronger, when larger irregularities were introduced into the stimuli by displacing the snare drum events, compared to when the originally performed microtiming pattern magnitude was increased. In other words: the few fixed snare displacements against a quantized background were more irritating to expert listeners than the flexible timing displacements occurring all over the actual performances.

The *Shift* of the bass track against the drums track did not have such an effect. Tests applied to the *Non-Experts* and *Swing* data were not significant; similarly, no effects were measured with respect to the *Entrainment* and *Enjoyment* data (tables and figures are omitted).

### 4.3. Discussion

The question of whether and how different microtiming patterns affect the emotional reactions of listeners cannot be conclusively answered from the data available. We found some evidence that *Expert* listeners reacted more irritatedly to the fixed snare displacements of the *Disturbance* series in *Funk* compared to scaled microtiming variations of the original performances. But this result is isolated, and it needs to be corroborated by further research.

Nevertheless, the data offers clear pointers why the question at hand could not be fully answered: we observe that the independent variable (*Density-adjusted STD*) of the *Disturbance* and *Shift* pattern series showed considerably smaller ranges in comparison to the scaled original performance patterns. Under the assumption that microtiming magnitude is adequately measured by *Density-adjusted STD*, the microtiming deviations of the *Disturbance* and *Shift* series would need to be expanded in order to compare the patterns on an equal footing. Specifically, the maximum displacements in *Funk* would have to be augmented to 35 ms for the *Disturbance* pattern, and the *Shift* would need to be augmented to 34 ms in order to match at the *Density-adjusted STD* value of the original *Funk* performance. The deviations within the *Swing* stimuli would need to be augmented to 69 ms (*Disturbance*) and 59 ms (*Shift*) in order to attain the *Density-adjusted STD* value of the original performance (note that these magnitudes are fairly close to those used by Matsushita and Nomura (2016)). A new experiment could clarify the effect of different microtiming patterns by adjusting the *Density-adjusted STD* to comparable magnitudes.

## 5. Conclusions

This paper's *Study A* replicated results from previous research. It showed that, on one hand, the mutual displacement of the bass track against the drums track by a magnitude up to 24 ms (*Shift*) did not have a significant effect on listeners' groove experiences (thus solidifying the main result of Butterfield, 2010). On the other hand, the study showed that displacing the snare drum track alone against the other tracks (*Disturbance*) affected listeners' groove experience negatively (thus it confirmed a core finding of Frühauf et al., 2013). Overall, we could confirm that not only the magnitude of microtiming deviations seems to matter, but also which layers of a musical fabric were affected: displacements between instrumental parts played by the same musician had a negative effect, whereas displacements between parts played by different musicians had no effect.

The effect of the *Disturbance* manipulation measured in *Study A* was considerably smaller than the effect found by Frühauf et al. (2013). This may have had several reasons: firstly, *Study A* had a flaw in the experimental setup that invalidated the responses to the quantized stimuli. This loss of data affected the statistical power of the experiment adversely. Secondly, the musical patterns of the stimuli in *Study A* differed considerably from those in Frühauf et al. (2013). The latter used a simple, straightforward rock music drum pattern, in which the snare drum played the quintessential backbeat and nothing else. In the present paper, both the swing and funk stimuli present a more complicated pattern, where the snare drum may appear on different, more or less metrically important positions. Attention research claims that irregular signals are easier to detect, when they are perceived against a regular background (Scerbo et al., 1986; Bregman, 1999; Parasuraman, 2000; Helton et al., 2005; Dalton et al., 2007; Winkler et al., 2009), compared to an irregular background. The pattern presented in Frühauf et al. (2013) represents a regular background, whereas the music used in this paper's *Study A* are rhythmically far more complicated and irregular, and this might have affected both the detectability and emotional impact of the microtiming displacements.

*Study B* explored different ways of measuring the magnitude of microtiming deviations in music. Using data from an earlier study (Senn et al., 2016), it showed that emotional listener reactions aligned with each other across two select musical styles (*Funk* and *Swing*), when microtiming magnitude was measured as a proportion of the mean IOI, and rhythmic density is accounted for as a moderating factor. The result suggests that the *Density-adjusted STD* is potentially better suited than the simple *STD* or the *Tempo-adjusted STD* to measure and compare the magnitude of microtiming phenomena across a variety of musical situations. This finding resonates with results from research on just-noticeable differences in auditory perception (Friberg and Sundberg, 1995), sensori-motor synchronization (Madison, 2014) and attentional dynamics (Jones et al., 2006). Further studies are necessary to establish whether the result expands to other musical contexts beyond the two situations investigated in this paper.

Finally, *Study C* probed into the effects of different microtiming patterns on listeners' emotional response, using the *Density-adjusted STD* as common measure of microtiming magnitude. This inquiry yielded one marginal result only: it showed that music expert listeners reacted with more *Irritation* when the snare drum was displaced against a quantized background, compared to the widely distributed microtiming patterns present in the originally recorded performances. A collateral result is perhaps of greater importance: *Study C* showed that the artificially introduced microtiming deviations (*Shift, Disturbance*) were of far smaller magnitude than the displacements found in the original performances. This suggests that, in future research, the magnitudes of lab-created and performance-generated microtiming patterns need to be matched in order to be comparable.

Overlooking the corpus of groove studies concerned with microtiming, we may summarize that this branch of research has surprisingly few results to show for its efforts. This is what we know so far: stimuli with quantized timing receive high groove ratings, at least in the context of an experiment. We also know that groove ratings deteriorate, as microtiming deviations are expanded (Davies et al., 2013; Frühauf et al., 2013; Matsushita and Nomura, 2016; Senn et al., 2016). We further obtained some evidence that the groove ratings of stimuli with microtiming that arises in professional performance are on par with those elicited by stimuli with quantized timing (Senn et al., 2016) and that it is not necessarily the quantized stimuli that motivate the most intense body movement in listeners (Kilchenmann and Senn, 2015). Finally, Hofmann et al. (2017) suggested that tight microtiming patterns are preferred by listeners, compared to larger microtiming magnitudes.

These results offer little support to the claim of the *Theory of Participatory Discrepancies* that microtiming deviations are essential for groove. Rather, it led scholars to the opposite conclusion, namely that music should be played with as little microtiming as possible in order to have high groove—this opinion has been most prominently voiced by Merker (2014). This conclusion, however, is at odds with the firm belief of many musicians that microtemporal aspects are crucial for groove (Berliner, 1994; Monson, 1996; Doffman, 2008) and with the findings of Hove et al. (2007) that the presence of certain kinds of microtiming improves rhythmic precision in musicians.

In a recent article, Witek pointed out with respect to microtiming that “it seems to matter greatly *how* these rhythmic nuances are implemented,” and she observed that “the conditions under which microtiming is effective have been difficult to recreate in a laboratory setting” (Witek, 2016, p. 16, emphasis in the original). Taking into account relevant moderating factors bridging between “conditions” and using patterns from expert performance in empirical research may allow to reach beyond the lab situation and better capture the groove phenomenon. Up to now, most experimental studies investigated the effect fixed microtiming patterns, and manipulated them by scaling the *magnitude* of the deviations. But the effect of systematically varying the *patterning* of microtiming deviations while retaining the magnitude of the deviations has not yet been sufficiently approached. The methodological advantage of varying magnitude as an experimental variable consists in the fact that, being a quantity, magnitude can be manipulated along one single dimension. Varying patterns is inherently multidimensional, and it is difficult to imagine how systematic manipulations can be achieved.

Another yet unchartered territory in groove studies is related to the variability of microtiming patterns across the time of a performance. In the past, average microtiming variation profiles have been extracted from performed music (see examples in Fujii et al., 2011; Kilchenmann and Senn, 2011, 2015; Naveda et al., 2011; Hellmer and Madison, 2015; Hofmann et al., 2017). These patterns may well represent a systematic aspect of microtiming in a certain style or context. But they do not take the procedural aspect of music performance into account: the development of the music in time and the rhythmic interaction between players. According to Keil (1966, 1987, 1995, 2010) this procedural aspect is of great importance. Large's and Jones' *Theory of Dynamic Attending* (Large and Jones, 1999; Jones et al., 2006) might provide a solid conceptual basis for tackling process-related aspects of musical performance.

In recent years, research has moved on to discuss other musical aspects that promise to be relevant for groove, besides microtiming. *Beat salience, event density* and *pulse clarity* have been identified as possible sources for (or at least correlates of) groove (Madison et al., 2011; Stupacher et al., 2016). Several studies have shown that *syncopation* is associated with the experience of groove (Madison and Sioros, 2014; Sioros et al., 2014; Witek et al., 2014) and with body movement (Witek et al., 2017). The groove qualities of rhythmic patterns have also been discussed, albeit not yet empirically (Zbikowski, 2004; Danielsen, 2006; Witek, 2016). The diversification of groove studies, to include other factors than microtiming, is a fruitful and necessary development. There is currently no shared doctrine among scholars whether microtiming is relevant or not, but everybody will agree that microtiming cannot be the only relevant aspect for groove, and that there are innumerable other routes to explore.

## Ethics statement

This study was carried out in accordance with the recommendations of the Ethics Commission of the Canton of Lucerne. All subjects gave written informed consent in accordance with the Declaration of Helsinki. The protocol was approved by the Ethics Committee of the Canton of Lucerne.

## Author contributions

Conceived and designed the experiments, wrote the paper, and designed the *EAG* questionnaire: OS, LK, RvG, and CB. Conducted the studio recording session and performed the experiments: LK. Validated the *EAG* questionnaire: RvG. Analyzed the data: OS.

### Conflict of interest statement

The authors declare that the research was conducted in the absence of any commercial or financial relationships that could be construed as a potential conflict of interest.

## Footnotes

^1^LARA (version 2.6.3) can be downloaded for free on www.hslu.ch/lara (26 September, 2017).

## References

[B1] Abel S. M. (1972). Discrimination of Temporal Gaps. J. Acoust. Soc. Am. 52, 519–524. 10.1121/1.1913139

[B2] Backs R. W. Silva S. P. D. Han K. (2005). A comparison of younger and older adults' self-assessment manikin ratings of affective pictures. Exp. Aging Res. 31, 421–440. 10.1080/03610730500206808 16147461

[B3] Baron R. M. Kenny D. A. (1986). The moderator-mediator variable distinction in social psychological research: conceptual, strategic, and statistical considerations. J. Pers. Soc. Psychol. 51, 1173–1182. 10.1037/0022-3514.51.6.1173 3806354

[B4] Benadon F. Zanette D. H. (2015). A corpus analysis of rubato in Bach's C Major Prelude, WTC I. Music Perform. Res. 7, 1–26.

[B5] Bengtsson I. (1974). Empirische Rhythmusforschung in Uppsala. Hamburger Jahrbuch Musikwissenschaft 1, 195–219.

[B6] Bengtsson I. Gabrielsson A. (1977). Rhythm Research in Uppsala. Uppsala: Musical Academy.

[B7] Bengtsson I. Gabrielsson A. (1980). Methods for analysing performance of musical rhythm. Scand. J. Psychol. 21, 257–268. 10.1111/j.1467-9450.1980.tb00369.x

[B8] Beran J. Mazzola G. (2000). Timing microstructure in Schumann's Träumerei as an expression of harmony, rhythm, and motivic structure in music performance. Comput. Math. Appl. 39, 99–130. 10.1016/S0898-1221(00)00049-3

[B9] Berliner P. F. (1994). Thinking in Jazz: The Infinite Art of Improvisation. Chicago, IL: University of Chicago Press.

[B10] Bradley M. M. Lang P. J. (1994). Measuring emotion: the self-assessment manikin and the semantic differential. J. Behav. Ther. Exp. Psychiatry 25, 49–59. 10.1016/0005-7916(94)90063-9 7962581

[B11] Bregman A. S. (1999). Auditory Scene Analysis: The Perceptual Organization of Sound. Cambridge, MA: MIT Press.

[B12] Butterfield M. W. (2010). Participatory discrepancies and the perception of beats in jazz. Music Percept. 27, 157–176. 10.1525/mp.2010.27.3.157

[B13] Cabanac M. (2002). What is emotion? Behav. Process. 60, 69–83. 10.1016/S0376-6357(02)00078-5 12426062

[B14] Camara G. S. (2016). Swing in Early Funk and Jazz-Funk (1967-1971): Micro-Rhythmic and Macro-Structural Investigations. MA Thesis, Universitetet i Oslo, Oslo.

[B15] Cheng E. Chew E. (2008). Quantitative analysis of phrasing strategies in expressive performance: computational methods and analysis of performances of unaccompanied Bach for solo violin. J. New Music Res. 37, 325–338. 10.1080/09298210802711660

[B16] Clarke E. F. (1982). Timing in the performance of Eric Satie's Vexations. Acta Psychol. 50, 1–19. 10.1016/0001-6918(82)90047-6

[B17] Cohen J. (1988). Statistical Power Analysis for the Behavioral Sciences, 2nd Edn. New York, NY: Psychology Press.

[B18] Cohen J. (2003). Applied Multiple Regression/Correlation Analysis for the Behavioral Sciences, 3rd Edn. Mahwah, NJ: Erlbaum.

[B19] Cook N. (1987). Structure and performance timing in Bach's C major prelude (WTCI): an empirical study. Music Anal. 6, 257–272. 10.2307/854205

[B20] Dalton B. H. Behm D. G. Kibele A. (2007). Effects of sound types and volumes on simulated driving, vigilance tasks and heart rate. Occupat. Ergon. 7, 153–168.

[B21] Danielsen A. (2006). Presence and Pleasure: The Funk Grooves of James Brown and Parliament. Middletown, CT: Wesleyan University Press.

[B22] Davies M. Madison G. Silva P. Gouyon F. (2013). The effect of microtiming deviations on the perception of groove in short rhythms. Music Percept. 30, 497–510. 10.1525/mp.2013.30.5.497

[B23] Dittmar C. Pfleiderer M. Müller M. (2015). Automated estimation of ride cymbal swing ratios in jazz recordings, in Proceedings of ISMIR 2015, Vol. 16 (Malaga), 271–277.

[B24] Dodson A. (2011). Expressive asynchrony in a recording of Chopin's prelude no. 6 in B minor by Vladimir de Pachmann. Music Theory Spect. 33, 59–64. 10.1525/mts.2011.33.1.59

[B25] Doffman M. R. (2008). Feeling the Groove: Shared Time and Its Meanings for Three Jazz Trios. PhD Thesis, The Open University, Milton Keynes.

[B26] Drake C. Botte M.-C. (1993). Tempo sensitivity in auditory sequences: evidence for a multiple-look model. Percept. Psychophys. 54, 277–286. 10.3758/BF03205262 8414886

[B27] Ehrlé N. Samson S. (2005). Auditory discrimination of anisochrony: influence of the tempo and musical backgrounds of listeners. Brain Cogn. 58, 133–147. 10.1016/j.bandc.2004.09.014 15878734

[B28] Fischinger T. (2009). Zur Psychologie des Rhythmus: Präzision und Synchronisation bei Schlagzeugern. Kassel: Kassel University Press.

[B29] Fraisse P. (1956). Les Structures Rhythmiques: Etude Psychologique. Brussels: Erasme.

[B30] Fraisse P. (1967). Le seuil différentiel de durée dans une suite régulière d'intervalles. L'année Psychologique 67, 43–49. 10.3406/psy.1967.27548 5601465

[B31] Friberg A. Bresin R. Sundberg J. (2006). Overview of the KTH rule system for musical performance. Adv. Cogn. Psychol. 2, 145–161. 10.2478/v10053-008-0052-x

[B32] Friberg A. Sundberg J. (1995). Time discrimination in a monotonic, isochronous sequence. J. Acoust. Soc. Am. 98, 2524–2531. 10.1121/1.413218

[B33] Friberg A. Sundström A. (2002). Swing ratios and ensemble timing in jazz performance: evidence for a common rhythmic pattern. Music Percept. 19, 333–349. 10.1525/mp.2002.19.3.333

[B34] Frühauf J. Kopiez R. Platz F. (2013). Music on the timing grid: the influence of microtiming on the perceived groove quality of a simple drum pattern performance. Music. Sci. 17, 246–260. 10.1177/1029864913486793

[B35] Fujii S. Hirashima M. Kudo K. Ohtsuki T. Nakamura Y. Oda S. (2011). Synchronization error of drum kit playing with a metronome at different tempi by professional drummers. Music Percept. 28, 491–503. 10.1525/mp.2011.28.5.491

[B36] Gabrielsson A. (1974). Performance of rhythm patterns. Scand. J. Psychol. 15, 63–72. 10.1111/j.1467-9450.1974.tb00557.x

[B37] Gomez P. Danuser B. (2007). Relationships between musical structure and psychophysiological measures of emotion. Emotion 7, 377–387. 10.1037/1528-3542.7.2.377 17516815

[B38] Halpern A. R. Darwin C. J. (1982). Duration discrimination in a series of rhythmic events. Percept. Psychophys. 31, 86–89. 10.3758/BF03206204 7070941

[B39] Hasty C. F. (1997). Meter as Rhythm. New York, NY: Oxford University Press.

[B40] Hellmer K. Madison G. (2015). Quantifying microtiming patterning and variability in drum kit recordings. Music Percept. 33, 147–162. 10.1525/mp.2015.33.2.147

[B41] Helton W. S. Hollander T. D. Warm J. S. Matthews G. Dember W. N. Wallaart M.. (2005). Signal regularity and the mindlessness model of vigilance. Brit. J. Psychol. 96, 249–261. 10.1348/000712605X38369 15969834

[B42] Hibi S. (1983). Rhythm perception in repetitive sound sequence. J. Acoust. Soc. Jpn. 4, 83–95. 10.1250/ast.4.83

[B43] Hirsh I. J. Monahan C. B. Grant K. W. Singh P. G. (1990). Studies in auditory timing: 1. Simple patterns. Percept. Psychophys. 47, 215–226. 10.3758/BF03204997 2326145

[B44] Hodson R. D. (2007). Interaction, Improvisation, and Interplay in Jazz. New York, NY: Routledge.

[B45] Hofmann A. Wesolowski B. C. Goebl W. (2017). The tight-interlocked rhythm section: production and perception of synchronisation in jazz trio performance. J. New Music Res. 46, 1–13. 10.1080/09298215.2017.1355394 PMC5706983 29238387

[B46] Hong J.-L. (2003). Investigating expressive timing and dynamics in recorded cello performances. Psychol. Music 31, 340–352. 10.1177/03057356030313006

[B47] Hoopen G. T. Hartsuiker R. Sasaki T. Nakajima Y. Tanaka M. Tsumura T. (1995). Auditory isochrony: time shrinking and temporal patterns. Perception 24, 577–593. 10.1068/p240577 7567431

[B48] Hove M. J. Krumhansl C. L. Keller P. E. (2007). Sensorimotor synchronization with chords containing tone-onset asynchronies. Percept. Psychophys. 69, 699–708. 10.3758/BF03193772 17929693

[B49] Huberty C. J. Morris J. D. (1989). Multivariate analysis versus multiple univariate analyses. Psychol. Bull. 105, 302–308. 10.1037/0033-2909.105.2.302

[B50] Janata P. Tomic S. T. Haberman J. M. (2012). Sensorimotor coupling in music and the psychology of the groove. J. Exp. Psychol. 141, 54–75. 10.1037/a0024208 21767048

[B51] Jones M. R. Puente J. Johnston H. M. (2006). Effects of auditory pattern structure on anticipatory and reactive attending. Cogn. Psychol. 53, 59–96. 10.1016/j.cogpsych.2006.01.003 16563367

[B52] Keil C. (1966). Motion and feeling through music. J. Aesthet. Art Criticism 24, 337–349. 10.2307/427969

[B53] Keil C. (1987). Participatory discrepancies and the power of music. Cult. Anthropol. 2, 275–283. 10.1525/can.1987.2.3.02a00010

[B54] Keil C. (1995). The theory of participatory discrepancies: a progress report. Ethnomusicology 39, 1–19. 10.2307/852198

[B55] Keil C. (2010). Defining “groove”. PopScriptum 11, 1–5.

[B56] Kilchenmann L. Senn O. (2011). Play in time, but don't play time: analyzing timing profiles of drum performances, in Proceedings of the International Symposium on Performance Science 2011 (Utrecht: AEC), 593–598.

[B57] Kilchenmann L. Senn O. (2015). Microtiming in swing and funk affects the body movement behavior of music expert listeners. Front. Psychol. 6:1232. 10.3389/fpsyg.2015.01232 26347694 PMC4542135

[B58] Large E. W. Jones M. R. (1999). The dynamics of attending: how people track time-varying events. Psychol. Rev. 106, 119–159. 10.1037/0033-295X.106.1.119

[B59] London J. (2004). Hearing in Time: Psychological Aspects of Musical Meter. Oxford: Oxford University Press.

[B60] Lunney H. W. M. (1974). Time as heard in speech and music. Nature 249:592. 10.1038/249592a0 4834087

[B61] MacRitchie J. (2011). Elucidating Musical Structure through Empirical Measurement of Performance Parameters. PhD, University of Glasgow.

[B62] Madison G. (2014). Sensori-motor synchronisation variability decreases as the number of metrical levels in the stimulus signal increases. Acta Psychol. 147, 10–16. 10.1016/j.actpsy.2013.10.002 24268879

[B63] Madison G. Gouyon F. Ullén F. Hörnström K. (2011). Modeling the tendency for music to induce movement in humans: first correlations with low-level audio descriptors across music genres. J. Exp. Psychol. 37, 1578–1594. 10.1037/a0024323 21728462

[B64] Madison G. Sioros G. (2014). What musicians do to induce the sensation of groove in simple and complex melodies, and how listeners perceive it. Front. Psychol. 5:894. 10.3389/fpsyg.2014.00894 25191286 PMC4137755

[B65] Matsushita S. Nomura S. (2016). The asymmetrical influence of timing asynchrony of bass guitar and drum sounds on groove. Music Percept. 34, 123–131. 10.1525/mp.2016.34.2.123

[B66] Merker B. (2014). Groove or swing as distributed rhythmic consonance: introducing the groove matrix. Front. Hum. Neurosci. 8:454. 10.3389/fnhum.2014.00454 25002843 PMC4066702

[B67] Michon J. A. (1964). Studies on subjective duration: I. Differential sensitivity in the perception of repeated temporal intervals. Acta Psychol. 22, 441–450. 10.1016/0001-6918(64)90032-0 14286022

[B68] Moelants D. (2011). The performance of notes ingales: the influence of tempo, musical structure, and individual performance style on expressive timing. Music Percept. 28, 449–460. 10.1525/mp.2011.28.5.449

[B69] Monson I. (1996). Saying Something: Jazz Improvisation and Interaction. Chicago, IL: University of Chicago Press.

[B70] Nakajima Y. (1987). A model of empty duration perception. Perception 16, 485–520. 10.1068/p160485 3444731

[B71] Nakajima Y. Hoopen G. T. Hilkhuysen G. Sasaki T. (1992). Time-shrinking: a discontinuity in the perception of auditory temporal patterns. Percept. Psychophys. 51, 504–507. 10.3758/BF03211646 1594440

[B72] Naveda L. Gouyon F. Guedes C. Leman M. (2011). Microtiming patterns and interactions with musical properties in samba music. J. New Music Res. 40, 225–238. 10.1080/09298215.2011.603833

[B73] Nordmark J. O. (1968). Mechanisms of frequency discrimination. J. Acoust. Soc. Am. 44, 1533–1540. 10.1121/1.1911293 5702028

[B74] Parasuraman R. (2000). The Attentive Brain. A Bradford book. Cambridge: MIT Press.

[B75] Philip R. (2004). Performing Music in the Age of Recording. New Haven, CT: Yale University Press.

[B76] Rasch R. A. (1988). Timing and synchronization in ensemble performance, in Generative Processes in Music: The Psychology of Performance, Improvisation, and Composition, ed Sloboda J. A. (Oxford: Clarendon Press), 70–90.

[B77] Repp B. H. (1992). Diversity and commonality in music performance: an analysis of timing microstructure in Schumann's Träumerei. J. Acoust. Soc. Am. 92, 2546–2568. 10.1121/1.404425 1479119

[B78] Repp B. H. (1995). Quantitative effects of global tempo on expressive timing in music performance: some perceptual evidence. Music Percept. 13, 39–57. 10.2307/40285684

[B79] Repp B. H. (1997). Expressive timing in a Debussy Prelude: a comparison of student and expert pianists. Music. Sci. 1, 257–268. 10.1177/102986499700100206

[B80] Repp B. H. (1998a). Obligatory ‘expectations’ of expressive timing induced by perception of musical structure. Psychol. Res. 61, 33–43. 10.1007/s004260050011 9532960

[B81] Repp B. H. (1998b). Variations on a theme by Chopin: relations between perception and production of timing in music. J. Exp. Psychol. Hum. Percept. Perform. 24, 791–811. 10.1037/0096-1523.24.3.791 9627417

[B82] Repp B. H. (2005). Sensorimotor synchronization: a review of the tapping literature. Psychon. Bull. Rev. 12, 969–992. 10.3758/BF03206433 16615317

[B83] Repp B. H. Su Y.-H. (2013). Sensorimotor synchronization: a review of recent research (2006–2012). Psychon. Bull. Rev. 20, 403–452. 10.3758/s13423-012-0371-2 23397235

[B84] Repp B. H. Windsor W. L. Desain P. (2002). Effects of tempo on the timing of simple musical rhythms. Music Percept. 19, 565–593. 10.1525/mp.2002.19.4.565

[B85] Sasaki T. Nakajima Y. Hoopen G. T. (1998). Categorical rhythm perception as a result of unilateral assimilation in time-shrinking. Music Percept. 16, 201–222. 10.2307/40285787

[B86] Scerbo M. W. Warm J. S. Fisk A. D. (1986). Event asynchrony and signal regularity in sustained attention. Curr. Psychol. 5, 335–343. 10.1007/BF02686601

[B87] Seeger C. (1958). Prescriptive and descriptive music-writing. Music. Q. 44, 184–195. 10.1093/mq/XLIV.2.184

[B88] Senn O. Kilchenmann L. Camp M.-A. (2009). Expressive timing: Martha Argerich plays Chopins Prelude op. 28/4 in E minor, in Proceedings of the International Symposium on Performance Science 2009, eds Williamon A. Pretty S. Buck R. (Utrecht: AEC), 107–112.

[B89] Senn O. Kilchenmann L. Camp M.-A. (2012). A turbulent acceleration into the stretto: Martha Argerich plays Chopin's Prelude op. 28/4 in E minor. Dissonance 120, 31–35.

[B90] Senn O. Kilchenmann L. von Georgi R. Bullerjahn C. (2016). The effect of expert performance microtiming on listeners' experience of groove in swing or funk music. Front. Psychol. 7:1487 10.3389/fpsyg.2016.01487 27761117 PMC5050221

[B91] Sioros G. Miron M. Davies M. Gouyon F. Madison G. (2014). Syncopation creates the sensation of groove in synthesized music examples. Front. Psychol. 5:1036. 10.3389/fpsyg.2014.01036 25278923 PMC4165312

[B92] Stupacher J. Hove M. J. Janata P. (2016). Audio features underlying perceived groove and sensorimotor synchronization in music. Music Percept. 33, 571–589. 10.1525/mp.2016.33.5.571

[B93] Sundberg J. (2003). Attempts to reproduce a pianist's expressive timing with Director Musices performance rules. J. New Music Res. 32, 317–325. 10.1076/jnmr.32.3.317.16867

[B94] Thomas K. (2007). Just noticeable difference and tempo change. J. Sci. Psychol. 2, 14–20.

[B95] Šidàk Z. (1967). Rectangular confidence regions for the means of multivariate normal distributions. J. Am. Stat. Assoc. 62, 626–633.

[B96] Winkler I. Denham S. L. Nelken I. (2009). Modeling the auditory scene: predictive regularity representations and perceptual objects. Trends Cogn. Sci. 13, 532–540. 10.1016/j.tics.2009.09.003 19828357

[B97] Witek M. A. G. (2016). Filling in: syncopation, pleasure and distributed embodiment in groove. Music Anal. 36, 138–160. 10.1111/musa.12082

[B98] Witek M. A. G. Clarke E. F. Wallentin M. Kringelbach M. L. Vuust P. (2014). Syncopation, body-movement and pleasure in groove music. PLoS ONE 9:e94446. 10.1371/journal.pone.0094446 24740381 PMC3989225

[B99] Witek M. A. G. Popescu T. Clarke E. F. Hansen M. Konvalinka I. Kringelbach M. L.. (2017). Syncopation affects free body-movement in musical groove. Exp. Brain Res. 235, 995–1005. 10.1007/s00221-016-4855-6 28028583

[B100] Zbikowski L. M. (2004). Modelling the groove: conceptual structure and popular music. J. R. Music. Assoc. 129, 272–297. 10.1093/jrma/129.2.272

